# Active Transport and Diffusion Barriers Restrict Joubert Syndrome-Associated ARL13B/ARL-13 to an Inv-like Ciliary Membrane Subdomain

**DOI:** 10.1371/journal.pgen.1003977

**Published:** 2013-12-05

**Authors:** Sebiha Cevik, Anna A. W. M. Sanders, Erwin Van Wijk, Karsten Boldt, Lara Clarke, Jeroen van Reeuwijk, Yuji Hori, Nicola Horn, Lisette Hetterschijt, Anita Wdowicz, Andrea Mullins, Katarzyna Kida, Oktay I. Kaplan, Sylvia E. C. van Beersum, Ka Man Wu, Stef J. F. Letteboer, Dorus A. Mans, Toshiaki Katada, Kenji Kontani, Marius Ueffing, Ronald Roepman, Hannie Kremer, Oliver E. Blacque

**Affiliations:** 1 School of Biomolecular and Biomedical Science, University College Dublin, Belfield, Dublin, Ireland; 2 Department of Otorhinolaryngology, Radboud University Medical Center, Nijmegen, The Netherlands; 3 Nijmegen Centre for Molecular Life Sciences, Radboud University Medical Center, Nijmegen, The Netherlands; 4 Donders Institute for Brain, Cognition and Behaviour, Radboud University Medical Center, Nijmegen, The Netherlands; 5 Division of Experimental Ophthalmology and Medical Proteome Center, Center of Ophthalmology, University of Tübingen, Tübingen, Germany; 6 Department of Human Genetics, Radboud University Medical Center, Nijmegen, The Netherlands; 7 Institute for Genetic and Metabolic Disease, Radboud University Medical Center, Nijmegen, The Netherlands; 8 Department of Physiological Chemistry, Graduate School of Pharmaceutical Sciences, University of Tokyo, Bunkyo-ku, Tokyo, Japan; 9 Berlin Institute for Medical Systems Biology (BIMSB) at Max-Delbrück-Center for Molecular Medicine (MDC), Berlin, Germany; 10 Research Unit Protein Science, Helmholtz Zentrum München, German Research Center for Environmental Health (GmbH), Neuherberg, Germany; Washington University School of Medicine, United States of America

## Abstract

Cilia are microtubule-based cell appendages, serving motility, chemo-/mechano-/photo- sensation, and developmental signaling functions. Cilia are comprised of distinct structural and functional subregions including the basal body, transition zone (TZ) and inversin (Inv) compartments, and defects in this organelle are associated with an expanding spectrum of inherited disorders including Bardet-Biedl syndrome (BBS), Meckel-Gruber Syndrome (MKS), Joubert Syndrome (JS) and Nephronophthisis (NPHP). Despite major advances in understanding ciliary trafficking pathways such as intraflagellar transport (IFT), how proteins are transported to subciliary membranes remains poorly understood. Using *Caenorhabditis elegans* and mammalian cells, we investigated the transport mechanisms underlying compartmentalization of JS-associated ARL13B/ARL-13, which we previously found is restricted at proximal ciliary membranes. We now show evolutionary conservation of ARL13B/ARL-13 localisation to an Inv-like subciliary membrane compartment, excluding the TZ, in many *C. elegans* ciliated neurons and in a subset of mammalian ciliary subtypes. Compartmentalisation of *C. elegans* ARL-13 requires a C-terminal RVVP motif and membrane anchoring to prevent distal cilium and nuclear targeting, respectively. Quantitative imaging in more than 20 mutants revealed differential contributions for IFT and ciliopathy modules in defining the ARL-13 compartment; IFT-A/B, IFT-dynein and BBS genes prevent ARL-13 accumulation at periciliary membranes, whereas MKS/NPHP modules additionally inhibit ARL-13 association with TZ membranes. Furthermore, *in vivo* FRAP analyses revealed distinct roles for IFT and MKS/NPHP genes in regulating a TZ barrier to ARL-13 diffusion, and intraciliary ARL-13 diffusion. Finally, *C. elegans* ARL-13 undergoes IFT-like motility and quantitative protein complex analysis of human ARL13B identified functional associations with IFT-B complexes, mapped to IFT46 and IFT74 interactions. Together, these findings reveal distinct requirements for sequence motifs, IFT and ciliopathy modules in defining an ARL-13 subciliary membrane compartment. We conclude that MKS/NPHP modules comprise a TZ barrier to ARL-13 diffusion, whereas IFT genes predominantly facilitate ARL-13 ciliary entry and/or retention via active transport mechanisms.

## Author Summary

Protruding from most cells surfaces is a hair-like extension called the primary cilium. This organelle functions as a cellular antenna, receiving physical and chemical signals such as light, odorants, and molecules that coordinate cell growth, differentiation and migration. Underscoring their importance, cilium defects underlie an expanding spectrum of diseases termed ciliopathies, characterised by wide-ranging symptoms such as cystic kidneys, blindness and bone abnormalities. A key question is how ciliary proteins are targeted to and retained within cilia. The best understood system is intraflagellar transport (IFT), thought to ferry proteins between the ciliary base and tip. Also, ciliopathy protein modules organise protein diffusion barriers at the ciliary base transition zone (TZ). Despite major advances, it remains poorly understood how proteins are targeted to cilia, and ciliary membrane subdomains in particular. Here, we investigated how Joubert syndrome-associated ARL13B/ARL-13 is compartmentalized at subciliary membranes. Using *C. elegans* nematodes and mammalian cell experimental systems, we uncovered differential requirements for sequence motifs, IFT and ciliopathy modules in regulating ARL-13 ciliary restriction, mobility and compartment length. Also, we provide essential insight into how IFT and ciliopathy-associated protein complexes and modules influence ciliary membrane protein transport, diffusion across the TZ, the integrity of the ciliary membrane, and subciliary protein composition.

## Introduction

Primary cilia are organized into specific subcompartments, defined by distinct ultrastructure, protein and lipid compositions, and include the basal body (BB), the adjacent transition zone (TZ), and axonemal regions consisting of doublet and singlet microtubules [1]. Ciliary subcompartments are important for the organelle's structural and functional properties. For example, BB transitional fibers anchor the cilium to the plasma membrane and serve as a docking site for ciliary transport machineries, and the TZ is thought to act as a ‘ciliary gate’ or diffusion barrier regulating protein access [2], [3]. Multiple proteins linked to ciliopathies such as Meckel-Gruber syndrome (MKS), nephronophthisis (NPHP), oral-facial digital syndrome (OFD) and Joubert syndrome (JS) are sequestered within specific ciliary subdomains. These include at least twenty MKS/NPHP/JS-associated proteins concentrated at the TZ, multiple ciliopathy proteins targeted specifically at the BB such as OFD1, and proteins such as NPHP2/INVS confined to a proximal ciliary subdomain called the Inversin compartment [3]–[5]. Functionally, many of these proteins regulate cilium-based signaling (e.g., via Sonic hedgehog and Wnt) that probably occurs at specific subciliary domains.

Targeting of proteins to cilia depends on intracellular transport mechanisms. The best studied is intraflagellar transport (IFT), an evolutionarily conserved motor protein-driven bidirectional motility of macromolecular assemblies along ciliary axonemes, essential for cilium formation and function (reviewed in [6], [7]). Anterograde IFT (base to tip) is driven predominantly by kinesin-2 motors, the canonical motor being heterotrimeric kinesin-II, whereas a cilium-specific cytoplasmic dynein complex powers retrograde IFT (tip to base). Associated with the motors and essential for IFT are IFT-B (∼14 proteins) and IFT-A (∼6 proteins) complexes. Proteins required for cilium biogenesis, maintenance and function are thought to be delivered to cilia by IFT and a handful of specific ‘cargos’ with IFT-like motility have been uncovered, including axonemal tubulin subunits, a transmembrane TRPV channel (OSM-9) and Polycystin 2 (PKD2) [8]–[10]. Additional putative cargos are Bardet-Biedl syndrome (BBS) proteins, which are known to regulate kinesin-2 motor (kinesin-II and homodimeric OSM-3/KIF17) association in *C. elegans* and flagellar export of signaling proteins in *Chlamydomonas* [11]–[14].

Compartmentalisation of ciliary proteins is heavily influenced by events at the ciliary base, with BB transitional fibers and TZ Y-links forming structural blocks to vesicle entry, and periciliary and TZ membranes thought to serve as diffusion barriers to membrane proteins (reviewed in [3], [15]). Although the molecular basis of these barriers is unclear, multiple ciliopathy proteins are implicated in regulating TZ ultrastructure and ciliary protein composition [3], [16]–[19]. In *C. elegans*, two genetically separable TZ modules with redundant ciliogenic functions are defined; an NPHP module consisting of NPHP1 and NPHP4, and an MKS module consisting of at least MKS1, B9D1/MKSR-1, B9D2/MKSR-2, MKS-2/TMEM16, MKS3, and MKS6/CC2D2A [19], [20]. At the periciliary/BB region, septin GTPases, which form ring and cage-like structures, prevent exchange of ciliary transmembrane proteins with non-ciliary pools [21], [22]. Ciliary ‘gating’ may also involve nuclear pore complex proteins and nucleocytoplasmic transport machinery, which localise at the ciliary base and in cilia, and are implicated in targeting proteins to the organelle [23]–[27]. Finally, the BB is where IFTA/B complexes, motors and cargo assemble into functional trains before moving into cilia.

We investigate ciliary protein transport in *C. elegans* sensory neurons. These highly polarised cells are ideal for ciliary transport studies since there is large spatial resolution between the various subcellular compartments and the primary cilium, which extends from distal tips of dendrites. Also, ciliary subcompartments are well defined. For example, amphid and phasmid channel cilia possess a degenerate basal body consisting only of transitional fibers, relatively long (∼1 µm) TZs, and bipartite axonemal structures consisting of doublet microtubules (middle segment) or singlet microtubules (distal segment) [28]. Most *C. elegans* cilia are environmentally exposed, relaying chemosensory, thermosensory and osmosensory signals. Many ciliary transport and ciliopathy genes are conserved in worms, and loss-of-function alleles are available for most of them. Importantly, unlike other systems, some resemblance of cilium structure remains in most IFT and ciliopathy gene mutants, thus allowing ciliary protein targeting to be investigated.

Despite major progress, the targeting and retention mechanisms regulating trafficking of cytosolic and membrane ciliary proteins are not well understood. For example, although IFT is an assumed driver of ciliary transport, only a handful of IFT cargos have been identified and there is evidence that a number of membrane proteins (e.g., PKD2) still localize to cilia in IFT disrupted cells, although ciliary abundance levels may be elevated [8], [9], [29], [30]. In particular, we know very little about how the various IFT subcomplexes and ciliopathy modules target proteins to specific ciliary membrane subdomains. To address these questions, this study focused on ARL13B, which is disrupted in a subset of Joubert syndrome patients (JBTS8; [31]). This membrane-associated small GTPase localises almost exclusively in cilia, and in *C. elegans*, the ARL-13 orthologue is further refined to a proximal ciliary subdomain [32]–[36]. ARL13B/ARL-13 is linked to a wide range of ciliary processes related to cilium formation, function and transport. These include the regulation of IFT, sonic hedgehog signaling, interneuronal migration, and chemosensation, as well as the entry, distribution and dynamics of ciliary signaling proteins [35]–[40].

Here we employed genetics, quantitative imaging, fluorescence recovery after photobleaching (FRAP) and affinity proteomics in nematode and cell culture models to investigate the mechanisms of ARL13B/ARL-13 transport and retention within a subciliary membrane domain. We show evolutionary conservation of ARL13B/ARL-13 localisation to an Inversin-like compartment and the requirement of RVVP and palmitoylation modification sequence motifs to prevent distal cilium and nuclear targeting in *C. elegans*. We also show distinct roles for IFT, BBS and TZ modules (MKS, NPHP) in regulating ARL-13 compartmentalization and diffusion across ciliary membranes. Finally, we determined the composition of human ARL13B complexes and uncovered robust biochemical associations with IFT complex B via IFT46 and IFT74 interactions. Overall, this study represents a comprehensive analysis of the transport mechanisms organizing the ARL13B/ARL-13 ciliary signaling subdomain, and provides important insight into how IFT and ciliopathy-associated protein complexes and modules influence ciliary transport and diffusion, the integrity of the ciliary membrane, and subciliary protein composition.

## Results

### ARL13B/ARL-13 is restricted to an Inv-like ciliary membrane subdomain, where it undergoes diffusion and IFT-like motility

**Figure 1:**
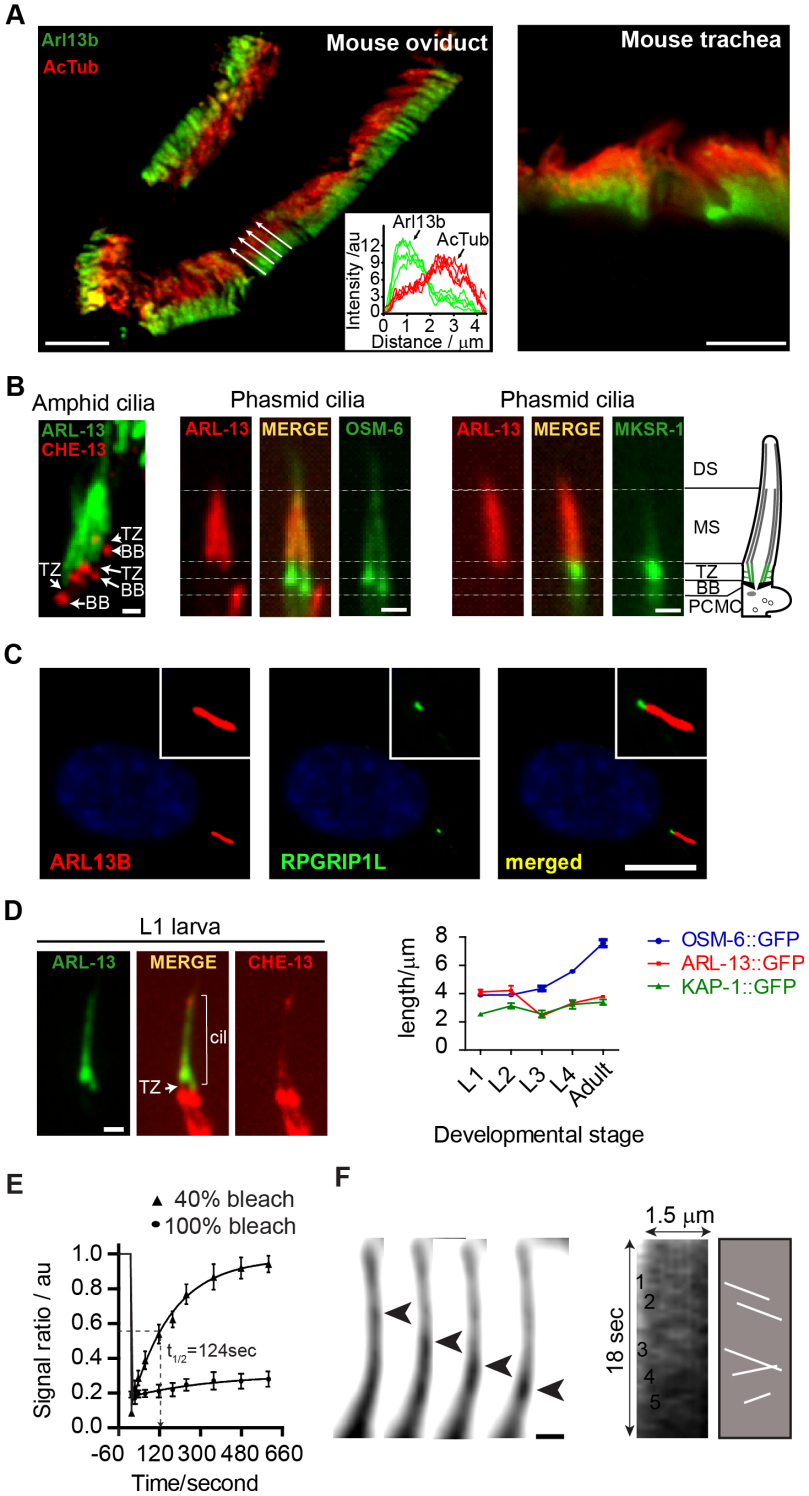
ARL-13/ARL13b localisation and mobility within an Inversin-like ciliary compartment. (**A**) Staining of mouse oviduct and tracheal tissue for endogenous Arl13b and acetylated tubulin shows proximal ciliary enrichment of Arl13b. Graph; line intensity profiles of Arl13b and acetylated tubulin (AcTub) signals from cilia denoted by white arrows. Bars; 5 µm (**B**) Co-expression of ARL-13::GFP with CHE-13/IFT57::mCherry, or ARL-13::tdTomato with either OSM-6::GFP or MKSR-1/B9D1::GFP, show that *C. elegans* ARL-13 is excluded from the transition zone (TZ). DS; distal segment. MS; middle segment. BB; basal body. PCMC; periciliary membrane compartment. Bars; 1 µm. (**C**) Staining of human hTERT-RPE1 cells shows that endogenous ARL13B does not colocalise with endogenous RPGRIP1L at the TZ. Bar; 10 µm (**D**) Phasmid cilia from L1 worms co-expressing ARL-13::GFP with CHE-13/IFT57::mCherry show that the ARL-13 compartment extends to the ciliary tips in young larva. Graph shows ARL-13::GFP, KAP-1::GFP (kinesin-II subunit) and OSM-6/IFT52::GFP ciliary compartment lengths in larval and adult stages of transgenic worms. Bar; 1 µm. (**E**) Fluorescence recovery after photobleaching (FRAP) curves after quenching 100%, or proximal-most 40%, of ARL-13::GFP ciliary signals in wild-type phasmid neurons. Signal ratio (au; arbitrary units) calculated from the average intensity of ARL-13 signal in the photobleached region compared to the non-photobleached region. All measurements are background subtracted and normalised to a pre-bleach ratio of 1.0. Each data point reports mean ± SEM. (**F**) Time-lapse images taken from a recording of an amphid channel cilium from worms expressing ARL-13::GFP show processive retrograde movement of an ARL-13::GFP-associated particle. Kymograph and associated schematic derived from one such recording show multiple moving anterograde and retrograde particles. Bar; 1 µm.

Previously we found that *C. elegans* ARL-13 localises to the proximal ciliary region of amphid (head) and phasmid (tail) channel cilia [35]. We now extend these findings, showing that endogenous mouse Arl13B is also sequestered to a proximal ciliary subdomain in oviduct and tracheal epithelial cells (**Figure 1A**). However, in agreement with published reports, Arl13b localises to the entire axoneme of proximal kidney cells (data not shown), thus Arl13b is excluded from distal regions of certain ciliary subtypes. Next we found that the *C. elegans* ARL-13 proximal ciliary domain in amphid and phasmid channel cilia corresponds to the middle segment (MS) and does not include the transition zone (TZ); specifically, ARL-13 is juxtaposed to MKSR-1/B9D1 at the TZ, and OSM-6/IFT52 basal body signals are separated from ARL-13 signals by an ∼1 µm ‘gap’, which corresponds to the TZ length (**Figure 1B**). Similarly in human retinal RPE1 cells, endogenous ARL13B is localised adjacent to TZ-localised MKS5/RPGRIP1L (**Figure 1C**). Together, these findings indicate evolutionary restriction in certain ciliary subtypes of ARL13B to a proximal ciliary compartment, excluding the TZ. This localisation is similar to that of Inversin (Inv) [4], [5].

We noticed that the ARL-13 compartment in phasmid cilia appeared longer in young worms versus adults. More detailed analysis revealed that at L1 stage, when phasmid cells are 12–15 hours old, ARL-13 decorated the entire cilium (**Figure 1D**; **Figure S1A**). At L2 stage, distal ciliary signals are dramatically reduced and by L3 stage, most worms show ARL-13 restriction to the proximal cilium (∼3 µm), which elongates slightly during development to adulthood (**Figure 1D**; **Figure S1A**). This developmental pattern was not observed for the overlapping heterotrimeric kinesin-II compartment [41], which by L1 stage is fully restricted to the proximal cilium (**Figure 1D**; **Figure S1A**). Also, ARL-13 is excluded from the phasmid TZs of all larval stages and the TZ ‘gap’ between ciliary ARL-13 and basal body CHE-13/IFT57 appears shorter in L1 larvae versus adults, suggesting TZ elongation as the worm ages (**Figure 1D**). Thus, at least in phasmid cilia, the ARL-13 ciliary membrane compartment undergoes post-embryonic remodelling.

Using a fluorescence recovery after photobleaching (FRAP) approach, we investigated if *C. elegans* ARL-13 is mobile at MS membranes. Photobleaching 100% of ARL-13::GFP ciliary signals resulted in almost no recovery, indicating limited or slow exchange with the dendritic compartment (**Figure 1E**). However, bleaching of ∼40% of ciliary signals resulted in relatively rapid signal recovery (t_1/2_ = 124 sec), concomitant with a reduction of the non-bleached ciliary signals (**Figure 1E** **; Figure S1B**). Recovery is wave-like, emanating from the non-bleached pool, and re-establishes pre-bleach uniform distribution of ARL-13 across the MS (**Figure S1B**). Thus, ARL-13 continuously exchanges at the MS membrane, but not between ciliary and dendritic membranes. These results are consistent with Arl13b FRAP dynamics in cultured cells [37].

Although it was previously reported by us and others that ARL-13 does not undergo IFT in adult worms [35], [36], bidirectionally moving particles containing ARL-13 can be detected in the amphid and phasmid channel cilia of young larval worms (**Figure 1F** **; Movie S1**). Although motility was more prominent in the distal cilium, movement was also detectable in proximal ciliary regions. For various technical reasons (photobleaching and immobilizing young larval worms), it was difficult to obtain many usable video microscopy-derived kymographs to measure motility rates. Nonetheless, for the particles we could measure, an anterograde speed of 0.65±0.09 µm.s^−1^ (n = 16) in phasmid cilia was determined, which is similar to reported MS anterograde IFT rates [41]. Thus, at least in developing or newly formed cilia, a proportion of ciliary ARL-13 appears to behave as IFT cargo.

### Sequence mechanisms restricting *C. elegans* ARL-13 to the middle segment membrane

**Figure 2:**
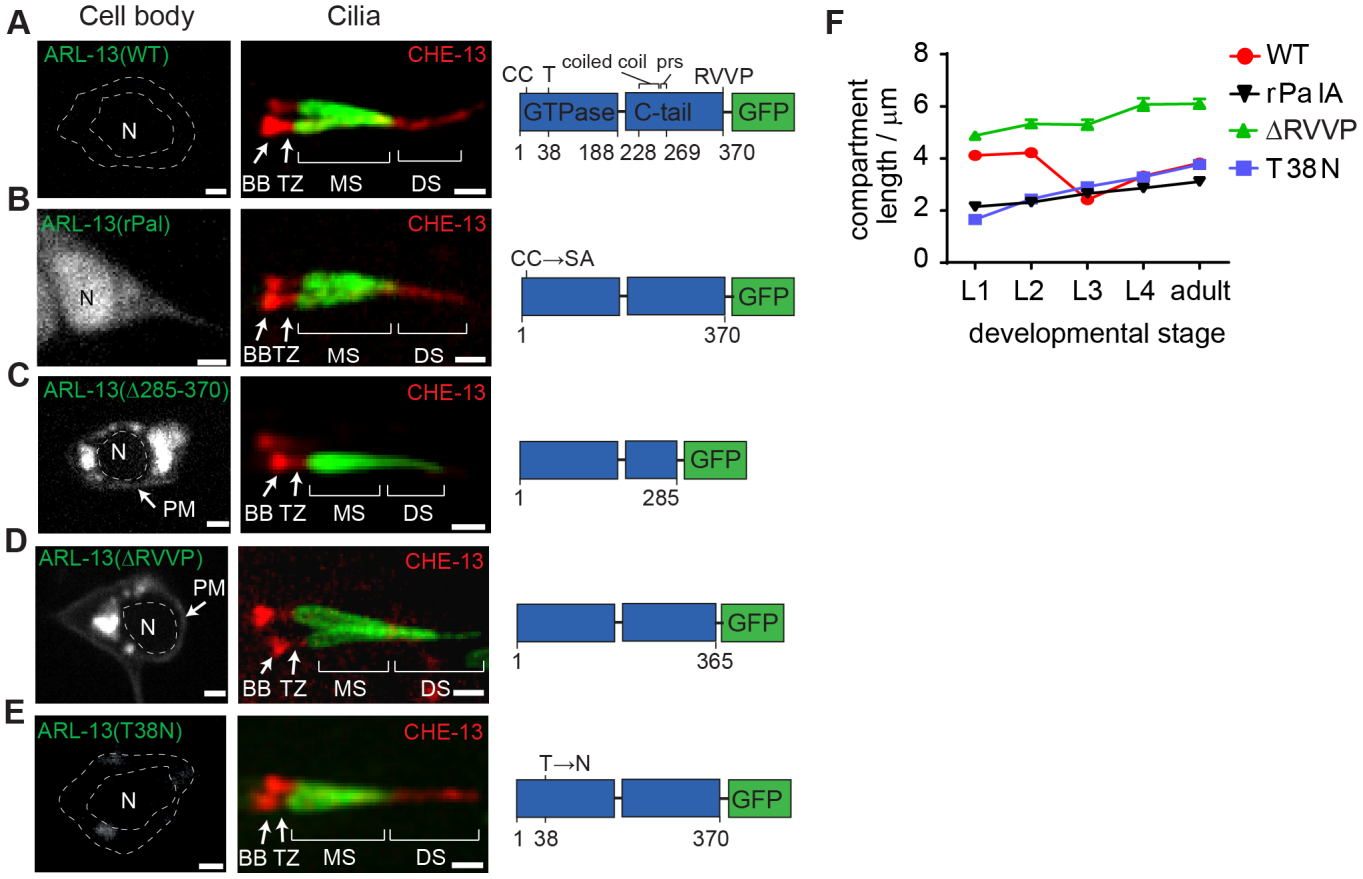
RVVP and palmitoylation modification motifs prevent targeting of ARL-13 to ciliary distal segments and the nucleus. (**A–E**) Shown are worms expressing a GFP-tagged ARL-13 sequence variant alone (left-hand images) or together with a CHE-13/IFT57::mCherry transgene (right-hand cilium images). Note that CHE-13 ciliary levels are highly reduced in Δ285–370 and ΔRVVP variants. rPal; replacement of N-terminal palmitoylation modification motif cysteines with Ser-Ala [35]. prs; proline-rich sequence. DS; distal segment. MS; middle segment. TZ; transition zone. BB; basal body. N; nucleus. Bars; 1 µm. (**F**) Plots of ARL-13 compartment length in phasmid cilia, at all larval stages, for worms expressing the indicated GFP-tagged ARL-13 variant or wild-type (WT) protein.

Previously we and others found that an N-terminal palmitoylation (Pal) modification motif and the disordered C-terminal tail restrict ARL-13 at ciliary membranes (**Figure 2A**) [35], [36]. Focusing now on the TZ, we find that these sequence elements are not required for ARL-13 TZ exclusion (**Figure 2B, C**). Instead, and agreeing with published findings [36], deletion of the C-terminal tail (Δ203–370 or Δ285–370) results in an elongated ARL-13 compartment spanning middle and distal segment membranes, although TZ exclusion was maintained (**Figure 2C, F**). Δ203–370 or Δ285–370 signals are also found at periciliary and plasma membranes (**Figure 2C**; data not shown). We mapped this function to a C-terminal RVVP motif, deletion of which caused a similarly expanded ARL-13 domain at all larval stages (**Figure 2D, F**). ΔRVVP and Δ285–370 (and Δ203–370) variants also showed punctate cell body accumulations (**Figure 2C, D**; data not shown), indicating a role for RVVP in early ARL-13 sorting, possibly similar to the TGN budding function of rhodopsin's VxPx motif [42]. However, ΔRVVP (and Δ203–370) cell body signals only partially colocalise with the TGN-marked SNARE protein, SYN-16 [43]; instead, most signals are juxtaposed, suggesting a transport block in cis-Golgi or another compartment (**Figure S2A**).

Next we found that Pal motif disruption caused nuclear targeting of ARL-13 in most sensory neurons (**Figure 2B**), suggesting that lipid modification inhibits a nuclear targeting pathway. This is consistent with a report showing that a 24 kDa C-terminal domain fragment (lacking the Pal motif) of mammalian Arl13b is nuclear targeted [37]. Although we could not find a nuclear import sequence in *C. elegans* ARL-13, Arl13b possesses a KRKK-like nuclear targeting signature in the C-terminal tail [37]. Thus, either the equivalent motif in ARL-13 is cryptic, or the mechanism of nuclear import is distinct. Consistent with reported findings for human ARL13B [33], a predicted GDP-locked variant (T38N) of ARL-13 was normally localised, indicating that GDP-GTP exchange is not required for restricting ARL-13 to middle segments (**Figure 2E, F**). We did observe, however, that unlike wild-type worms, the ARL-13 compartment of T38N and rPal-expressing worms did not extend to the ciliary tips in young larval L1 animals (**Figure 2F**); thus, GDP-GTP exchange and lipid modification of ARL-13 plays a subtle role during compartment morphogenesis.

Finally we investigated whether altered ARL-13 localisations disrupt its function. Consistent with normal localisations, an *arl-13(T38N)* transgene rescued the cilium integrity defect (measured by a dye-filling assay) of an *arl-13(tm2322)* mutant, indicating that GDP-locked ARL-13 is functional (**Figure S2B**). In contrast, Pal motif-disrupted *arl-13* constructs were previously reported by us to induce a mild dominant negative dye-filling (Dyf) phenotype, indicating disrupted function [35]. We now report a similar finding for the *arl-13(Δ285–370)* and *arl-13(ΔRVVP)* transgenes, which induce a fully penetrant dominant negative Dyf defect (**Figure S2C**; data not shown). To further investigate this defect, a CHE-13/IFT57 marker was used to stain cilia, which revealed that ARL-13(ΔRVVP)-expressing amphid cilia were abnormally dispersed and mis-positioned; furthermore, axonemal CHE-13 signals were reduced or absent, indicating an IFT defect (**Figure S2D**). These phenotypes were specific for *arl-13(ΔRVVP)*, and not found in *arl-13(rPal)* and *arl-13(T38N)* expressing worms (**Figure S2D**). Since *arl-13(tm2322)* worms also possess misplaced amphid cilia and reduced ciliary signals for IFT proteins [35], we conclude that the IFT and ciliogenesis-related functions of ARL-13 involves the C-terminal RVVP motif [35], [36], [38]. Furthermore, because reduced CHE-13 ciliary levels correlate with abnormal distal ciliary staining of ARL-13(ΔRVVP), IFT may prevent ARL-13 entry into distal segments.

These data show that a C-terminal RVVP motif restricts ARL-13 compartment size by preventing leakage into distal segments and suggest that palmitoylation regulates shuttling of ARL-13 between the ciliary membrane and the nucleus. Furthermore, our findings indicate that these motifs are required for the ciliogenic and IFT-related functions of ARL-13.

### IFT and MKS/NPHP modules are differentially required for ARL-13 localisation

Next, we questioned if ARL-13 ciliary targeting and restriction requires IFT-A/B, kinesin-2, IFT-dynein, BBS, MKS, NPHP or septin genes, all of which are associated with distinct aspects of ciliary protein transport. In simplified models, protein entry into cilia is facilitated by kinesin-2/IFT-B-driven anterograde IFT, whereas protein recycling from the ciliary tip involves IFT-dynein/IFT-A-driven retrograde IFT [6], [7], [44]. In reality, IFT is more complex, since some proteins require IFT-A for ciliary entry, and in *C. elegans* anterograde IFT assemblies, the kinesin-II motor is physically more closely connected with IFT-A versus IFT-B [12], [45], [46]. Ciliary gating at the TZ is thought to be facilitated by various MKS, NPHP and JS proteins, and in *C. elegans* MKS and NPHP modules play redundant roles in establishing the TZ [3], [16], [18], [19]. Finally, at least one mammalian septin (Sept2) is implicated in periciliary and TZ barrier functions, and BBS proteins regulate anterograde IFT and ciliary protein export [12], [13], [17], [21], [47]. We tested these ciliary targeting models from an ARL-13 viewpoint by investigating its localisation in more than 20 reduction-of-function mutants, most of which are nulls. Importantly, all mutants retain at least a short cilium, thus allowing ARL-13 compartmentalisation to be investigated. Of note, the septin family consists of two genes in worms; *unc-61* (class 1B) and *unc-59* (class 2B; includes mammalian *Sept2*) [48].

**Figure 3:**
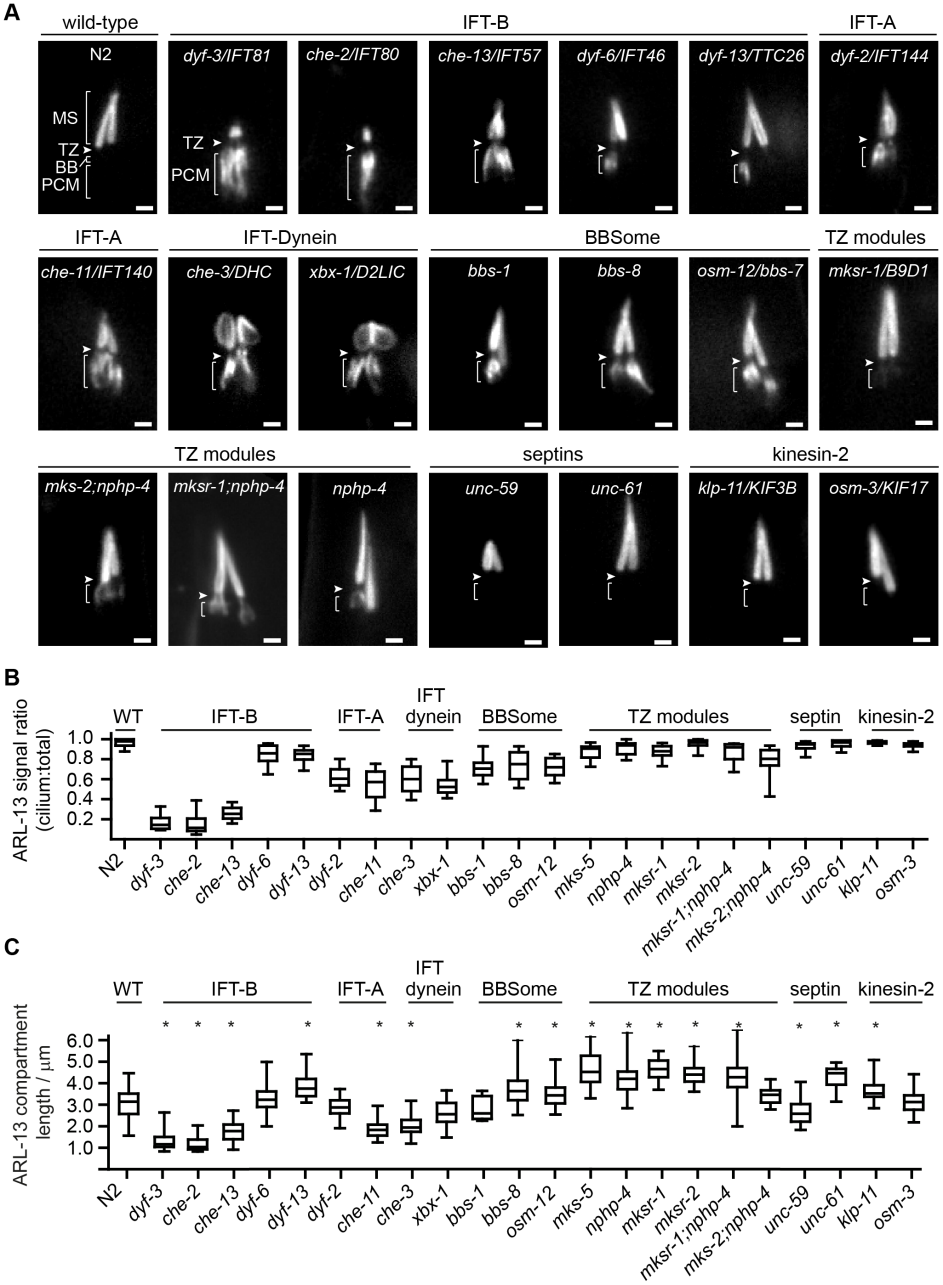
Differential requirements of ciliary transport and ciliopathy modules for ARL-13 ciliary compartmentalisation. (**A**) Phasmid cilia of worms expressing ARL-13::GFP in the indicated genotype. Arrowheads; transition zone (TZ). MS; middle segment. BB; basal body. PCM; periciliary membrane (bracket). Bars; 1 µm. (**B**) Box and whisker plot distribution of ARL-13 signal ratio in the cilium versus total (cilium+PCM). Measurements represent absolute signal intensities (arbitrary units) within both compartments, adjusted for background. (**C**) Box and whisker (min to max) distribution plots of ARL-13::GFP ciliary compartment length in phasmid neurons of the indicated mutant genotype. *p<0.001 (vs WT).

In WT worms, ARL-13 is found exclusively in cilia. However, in IFT-B, IFT-A, IFT-dynein and BBS gene mutants, ARL-13 was specifically mislocalised at the periciliary membrane (PCM) region and not in other regions of the sensory neuron **(** **Figure 3A**; **Figure S3A; Movies S2, S3, S4, S5, S6, S7, S8**). Quantification of signal intensities revealed that IFT-B gene mutants (except *dyf-6/IFT46* and *dyf-13/TTC26*) possessed the highest levels of PCM accumulation (relative to total ciliary levels), whereas IFT-A and IFT-dynein mutants displayed reduced PCM accumulation levels, with levels reduced further in BBS mutants (**Figure 3B**; statistics shown in **Table S1**). Although there was a trend between cilium length and accumulation levels (e.g., short cilia of IFT-B mutants correlate with high PCM accumulations), this correlation was not absolute. For example, *osm-3/KIF17* cilia are shorter than *bbs* mutant cilia [11], [49], yet the former displays no PCM accumulations (**Figure 3A, B**). In contrast to IFT-A/B/dynein/BBS mutants, single mutants with disrupted MKS (*mks-5*/RPGRIP1L, *mksr-1/B9D2*, *mksr-2/B9D1*) or NPHP (*nphp-4*) genes showed relatively weak ARL-13 PCM accumulations (**Figure 3A, B** **; Figure S3A; Table S1**). However, moderate levels of PCM staining was found in *mks-2/TMEM16;nphp-4* and *mksr-1;nphp-4* double mutants, indicating redundant functions for MKS and NPHP genes in regulating ARL-13 PCM exclusion (**Figure 3A, B** **; Figure S3A; Table S1**). We also observed punctate cell body spots of ARL-13 in *mks-2;nphp-4* and *mksr-1;nphp-4* worms, and to a lesser extent in *mks-5* worms, suggesting an additional role for TZ genes in an early ARL-13 sorting event (**Figure S3B**). Finally, ARL-13 localisation was unaffected in septin (*unc-59*/*61*) and kinesin-2 (*osm-3/KIF17* and *klp-11/KIF3B*) worms (**Figure 3A, B**; **Figure S3A**). Whilst the above phenotypes are reported for phasmid neurons, similar observations were made in amphid neurons.

Despite PCM accumulations, all examined mutants possess significant ARL-13 ciliary signals. To address if the ARL-13 domain is structurally altered in these worms, we investigated its length and proximo-distal patterning in phasmid cilia. In most IFT-A, IFT-B and IFT-dynein short cilia mutants, the ARL-13 compartment was correspondingly short and extended to the ciliary tip. However, in IFT-related mutants with cilia closer to wild-type lengths (*dyf-13/TTC26*, *klp-11/KIF3B*, *bbs-7* and *bbs-8*), the ARL-13 compartment was modestly elongated, suggesting a role for IFT in restricting ARL-13 subdomain length (**Figure 3C**). With the exception of occasional *che-3*, *xbx-1* and *dyf-13* animals, ARL-13 signals were not found at the TZ membrane of most IFT-related gene mutants, indicating these genes are typically not required for TZ exclusion of ARL-13 (**Figure 3A** **, Figure S3C**). Conversely, ARL-13 was frequently observed at the TZ region of most *mks-5* single mutants, as well as *mks-2;nphp-4* and *mksr-1;nphp-4* double mutants, demonstrating that MKS/NPHP modules regulate the composition of the TZ membrane (**Figure 3A** **; Figure S3C**). This TZ staining in MKS/NPHP mutants results in an elongated ARL-13 compartment, which in some worms appears to extend into distal ciliary regions also (**Figure 3C**).

Together, these data reveal overlapping and distinct roles for IFT and ciliopathy modules in defining the ARL-13 subciliary membrane compartment. IFT-A/B, IFT-dynein and BBS genes, and to a lesser extent TZ genes, prevent specific accumulation of ARL-13 at the PCM, whereas TZ genes inhibit ARL-13 association with the TZ membrane. In addition, IFT-related genes appear to restrict ARL-13 compartment length.

### An *in vivo* FRAP assay reveals distinct requirements for MKS, NPHP and IFT modules in regulating ARL-13 diffusion across the transition zone and in cilia

**Figure 4:**
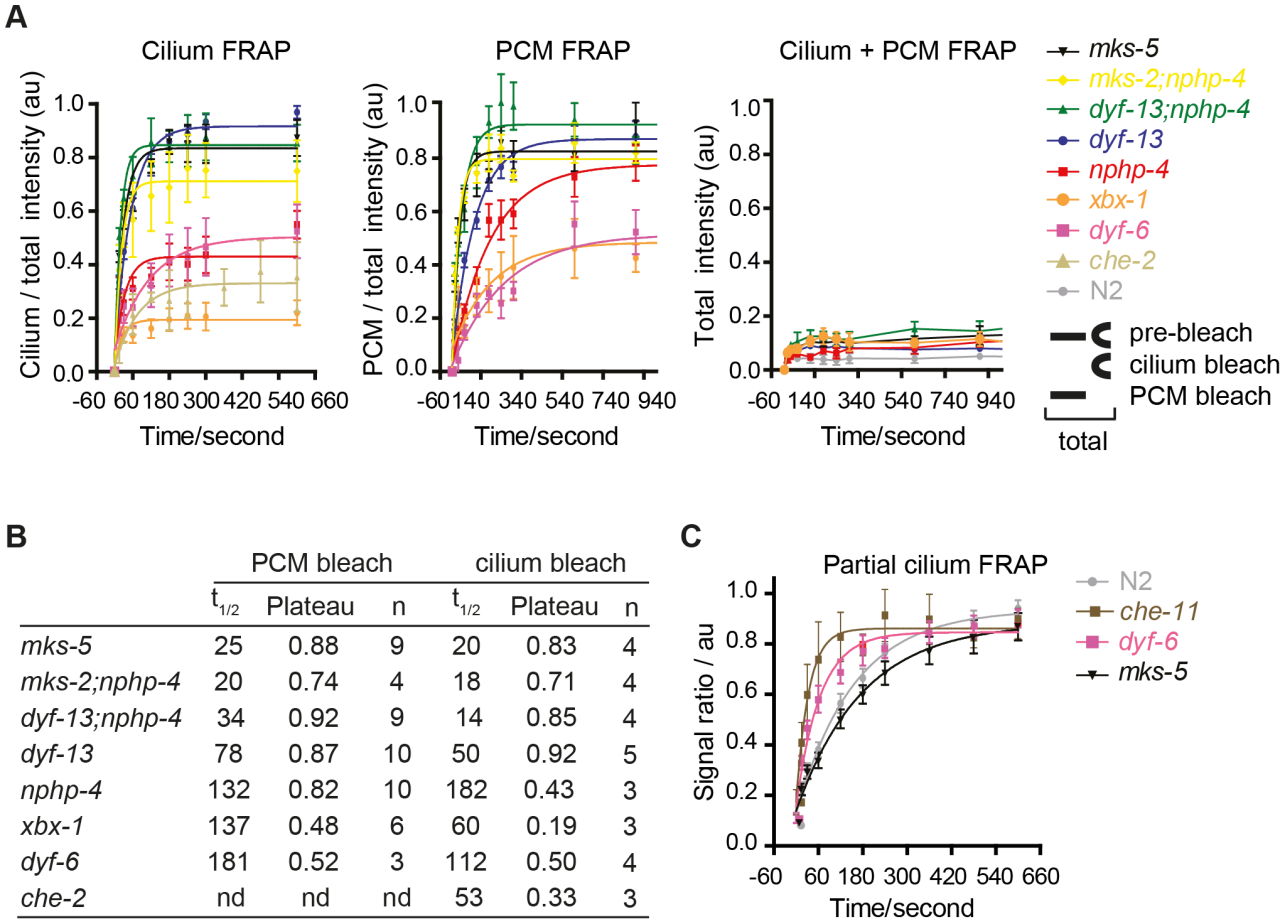
FRAP analysis of ARL-13 exchange dynamics between ciliary and periciliary membrane compartments in IFT and TZ gene mutants. (**A**) FRAP curves (background subtracted) derived from bleaching entire periciliary membrane (PCM), ciliary or PCM+ciliary ARL-13::GFP signals in phasmid cilia. For ease of comparison, pre-bleach ratios are normalised to 1.0, and bleach (t0 sec) time-points normalised to 0. (**B**) Half-time recoveries and plateau (maximum) recovery levels for graphs in (A). nd; not determined. (**C**) FRAP curves from photobleaching ∼40% of proximal-most ARL-13 signals in phasmid neurons of wild-type (n = 16), *dyf-6* (n = 14), *che-11* (n = 5) and *mks-5* (n = 11) mutant worms. Signal ratio (au; arbitrary units) calculated from the average intensity of ARL-13 signal in the photobleached region compared to the non-photobleached region.

One explanation for ARL-13 association with mutant periciliary and TZ membranes is that the TZ barrier is compromised, which causes ARL-13 to leak out of its compartment. An alternative non-mutually exclusive explanation is that active processes facilitating ARL-13 ciliary entry or retention might be defective. To test these hypotheses, we used our *in vivo* FRAP assay to investigate ARL-13 exchange kinetics between ciliary and PCM compartments in IFT and TZ gene mutants. Bleaching entire ciliary or PCM pools in *mks-5* or *mks-2;nphp-4* mutants lead to rapid signal recovery (t_1/2_<25 sec) back to pre-bleach ratios (**Figure 4A, B** **; Figure S4A, B; Movies S9, S10**). However, for the *nphp-4* mutant, PCM/ciliary exchange was relatively slow (t_1/2_ 132, 182 sec) and recovery plateaued well below pre-bleach ratios in cilium quenching experiments (**Figure 4A, B** **; Figure S4A, B; Movies S9, S10**). In these experiments, signals recovered from non-bleached pools (cilium or PCM) and not from other parts of the cell because no significant recovery was observed when both ciliary and periciliary ARL-13 signals were quenched (**Figure 4A** **, Figure S5A; Movie S11**); also, as bleached pools recovered, ARL-13 signal intensity at the non-quenched pools diminished (**Figure S4A**; data not shown). Thus, in *mks-5* and *mks-2;nphp-4* animals, but not *nphp-4* worms, the ciliary and PCM pools of ARL-13 are rapidly exchanging. In contrast, ARL-13 FRAP rates and recovery plateaus were much lower in most examined IFT mutants (*dyf-6/IFT46*, *che-2/IFT80* and *xbx-1/DLIC*), indicating these worms possess slow and low level diffusion between ciliary and PCM pools (**Figure 4A, B** **; Figure S4A, B; Movies S9, S10**; data not shown). The one exception was a *dyf-13/TTC26* (IFT-B) mutant, which displayed moderately fast FRAP rates (t_1/2_ 50, 78 sec) and high recovery levels (**Figure 4A, B** **; Figure S4A, B; Movies S9, S10**). Interestingly, *dyf-13;nphp-4* double mutants possessed faster bidirectional recovery kinetics than single mutants (t_1/2_ 14, 34 sec; p<0.01), possibly indicating partially redundant functions for these genes in regulating ARL-13 ciliary/PCM diffusion (**Figure 4A, B** **; Figure S4A, B; Movies S9, S10**). From these data we conclude that the TZ barrier to ARL-13 diffusion is disrupted in *mks-5*, *mks-2;nphp-4* and *dyf-13; nphp-4* mutants; thus the TZ and PCM accumulations in these worms are due to ARL-13 leakage out of its compartment. However, in most IFT and *nphp-4* mutants, the TZ diffusion barrier appears mostly intact, suggesting that any observed ARL-13 PCM accumulations are caused by defects in active transport driving ciliary entry and/or retention.

We also investigated if IFT and TZ modules regulate ARL-13 diffusion at the ciliary (middle segment; MS) membrane. Using the same FRAP assay described in Figure 1E, we found that photobleaching of ∼40% of proximal ARL-13 MS signals resulted in significantly faster recovery in *che-11/IFT140* (t_1/2_ = 23 sec; p<0.001) and *dyf-6/IFT46* (t_1/2_ = 43 sec; p = 0.05) worms, compared to wild-type worms (t_1/2_ = 124 sec) (**Figure 4C** **; Figure S5B**). Qualitatively similar results were observed when distal ARL-13 MS signals were bleached (**Movies S12**; compare WT vs *dyf-6*). In contrast, wild-type and *mks-5* worms (t_1/2_ = 116 sec; p = 0.17) possessed similar recovery rates (**Figure 4C** **)**. In all experiments the area, length and signal intensities of the photobleached MS region was comparable (**Figure S5C**). We conclude that IFT-A and IFT-B proteins (CHE-11, DYF-6), but not TZ-associated MKS-5, retard ARL-13 exchange kinetics at the MS membrane.

### Human ARL13B interacts with IFT-B subcomplexes via IFT46 and IFT74

**Figure 5:**
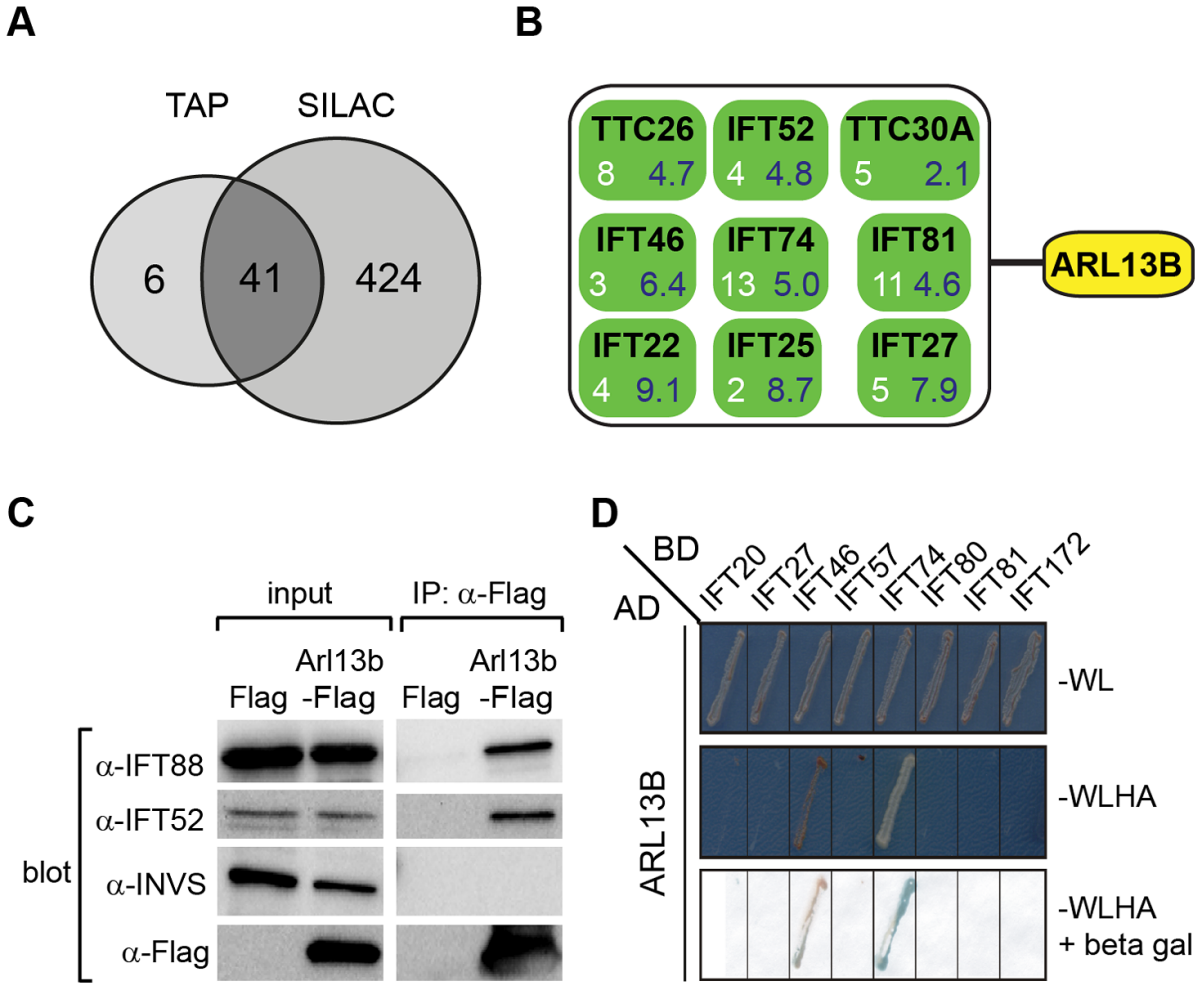
ARL13B associates with IFT-B complex via IFT46 and IFT74 interactions. (**A**) Number of proteins found to associate with SF (Strep-Flag)-tagged human ARL13B using TAP (tandem affinity chromatography) and SILAC (stable isotope labelling affinity chromatography) approaches in HEK293 cells. (**B**) ARL13B complexes possess IFT-B proteins. White number; average peptide count from 4 independent tandem-affinity purification experiments (details in Table 1). Blue number; average enrichment scores from 4 independent experiments employing a single purification/SILAC quantitative approach. TTC26 also known as DYF-13 in *C. elegans*, and TTC30A also known as IFT70 in *Chlamydomonas*. (**C**) Western blot from HEK293T cells transfected with Arl13b-Flag vector or Flag vector ‘mock’ control (Flag) showing that immunoprecipitated Flag-tagged ARL13B associates with IFT-B proteins (IFT88, IFT52) but not Inversin (INVS). (**D**) Dedicated yeast-two hybrid one-on-one analysis reveals direct interactions between human ARL13B and both IFT46 and IFT74. WL; minimal media lacking Trp and Leu. WLHA; minimal media lacking Trp, Leu, His and Ade.

To shed further light on ARL-13/ARL13B transport and compartmentalisation mechanisms, we employed affinity proteomics to identify the composition of human ARL13B complexes. ARL13B was fused with a Strep-Flag (SF) tandem affinity purification tag (TAP) [50] and expressed in HEK293T ciliated cells. Both N- and C-terminally SF-tagged ARL13B localised to the primary cilium of hTERT-RPE1 cells indicating that neither the TAP tag nor expression levels of this recombinant protein affects its subcellular localization (**Figure S6**). We first performed stringent two-step (tandem) affinity purifications (TAP), followed by mass-spectrometric identification of the co-precipitated proteins. Specific interactors were identified by comparing SF-tagged ARL13B precipitate profiles with control precipitates from cells expressing the SF tag alone. Two experiments were conducted for N-SF-ARL13B and one for C-SF-ARL13B. We also performed one experiment on cells expressing GDP-locked (T35N) ARL13B. Following removal of non-specific and obvious false positive proteins routinely found in TAP experiments (see methods section), these four experiments produced a final dataset of 47 proteins co-purifying with ARL13B (**Figure 5A** **;** **Table 1** **; Table S2**). Highly represented are components of the IFT complex B (IFT22, 25, 27, 46, 52, 70, 74, and 81) and one putative IFT-B protein (TTC26/DYF-13) (**Figure 5B** **;** **Table 1** **; Table S2**). Most of these proteins are suggested in *Chlamydomonas* to form a ∼500 kDa IFT-B core [51], [52]. Other interesting identified proteins were karyopherin beta proteins involved in nucleocytoplasmic transport, including 5 importins (IPO4/5/7/8/9), two exportins (XPO2/5) and transportin (TNPO1), as well as three ubiquitination-associated proteins, namely CAND1/2 (cullin-associated and neddylation-dissociated) and DCAF8 (DDB1 and CUL4 associated factor). Although some differences were observed between the N-SF-ARL13B and C-SF-ARL13B complexes, there was remarkable consistency across the experiments for IFT-B transport proteins, in terms of the specific proteins detected and the peptide counts obtained. Underscoring the specificity of the IFT-B associations, ARL13B complexes were devoid of IFT-A or BBSome proteins, and most proteins associated with MKS, NPHP or JS, the one exception being NPHP-linked Ataxin-10 [53]. Few if any differences were found in ARL13B(T35N) complexes (**Table S2**), indicating that ARL13B associates with IFT-B independent of GDP-GTP exchange.

**Table 1:** Identification of human ARL13B complex proteins.

Gene Symbol	Full name	TAP count	SILAC enrichment
ARL13B	ADP-ribosylation factor-like 13B	31.2	10.4
IFT74	intraflagellar transport 74	13.3	5.0
IFT81	intraflagellar transport 81	11.0	4.6
CAND2	cullin-associated and neddylation-dissociated 2	9.0	6.4
TTC26	tetratricopeptide repeat domain 26	7.7	4.7
DCAF8	DDB1 and CUL4 associated factor 8	6.8	8.0
IPO9	importin 9	6.7	7.5
TNPO1	transportin 1	6.3	3.7
FANCD2	Fanconi anemia, complementation group D2	6.2	6.2
TELO2	Telomere maintenance 2	5.5	5.0
IFT27	intraflagellar transport 27	5.3	7.9
CAND1	cullin-associated and neddylation-dissociated 1	5.3	nd
IPO4	importin 4	5.3	6.7
XPO2	exportin 2	5.2	nd
XPO5	exportin 5	5.0	2.6
TTC30A	tetratricopeptide repeat domain 30A	4.5	2.1
IFT22	RAS oncogene family-like 5.	4.3	9.1
ATXN10	ataxin 10	4.3	4.2
MMS19	MMS19 nucleotide excision repair homolog	4.3	4.5
PEX19	peroxisomal biogenesis factor 19	4.2	12.3
ARMC6	armadillo repeat containing 6	4.2	5.3
IPO8	importin 8	4.0	4.5
WDR17	WD repeat domain 17	3.7	5.3
IFT52	intraflagellar transport 52	3.5	4.8
HLA-C	major histocompatibility complex, class I, C	3.5	3.3
IFT46	intraflagellar transport 46	3.3	6.5
DHCR7	7-dehydrocholesterol reductase	2.8	3.1
ANKRD13A	ankyrin repeat domain 13A	2.3	4.2
GEMIN4	gem associated protein 4	2.3	4.7
IPO5	importin 5	2.3	2.2
CCDC47	coiled-coil domain containing 47	2.2	5.2
NPEPPS	aminopeptidase puromycin sensitive	2.2	2.6
MDH2	malate dehydrogenase 2	2.0	nd
CNP	2′,3′-cyclic nucleotide 3′ phosphodiesterase	2.0	4.1
IFT25	intraflagellar transport 25	1.8	8.7
ARL1	ADP-ribosylation factor-like 1	1.8	5.7
NUBP2	nucleotide binding protein 2	1.8	6.5
AAR2	AAR2 splicing factor homolog	1.8	nd
FASTKD1	FAST kinase domains 1	1.8	5.7
HEATR3	HEAT repeat containing 3	1.8	4.3
TTC27	tetratricopeptide repeat domain 27	1.8	5.3
S100A7	S100 calcium binding protein A7	1.7	nd
RPLP1	ribosomal protein, large, P1	1.7	2.7
HBB	hemoglobin, beta	1.7	nd
SAAL1	serum amyloid A-like 1	1.7	8.1
SUCLA2	succinate-CoA ligase, ADP-forming, beta subunit	1.7	4.4
IPO7	importin 7	1.5	2.4

Shown are average tandem affinity purification (TAP) peptide counts (4 independent experiments; details in Table S2) and SILAC enrichment factors (4 independent experiments; details in Table S3) of proteins co-immunoprecipitating with SF-tagged ARL13B(WT) or ARL13B(T35N). This list contains all 47 proteins uncovered by the TAP experiments, with an average peptide count >1.5. Note only 6 of these proteins were not detected (nd) using the more sensitive SILAC approach.

To validate the ARL13B complex components identified by TAP and to increase sensitivity in order to detect additional, more labile and transient ARL13B module components, we employed stable isotope labeling of amino acids in cell culture (SILAC), in combination with single step affinity purification and quantitative mass spectrometry. This allowed a quantitative comparison of SF-tagged ARL13B and the SF-TAP alone, or mutated SF-tagged ARL13B. After purification, eluates were combined and non-specific contaminants subtracted to detect potential interaction partners by their specific enrichment compared to the control [54]. This approach led to the enrichment of several hundred potential ARL13B(WT) complex components, including most of the proteins (41 out of 47) detected by TAP (**Figure 5A, B** **; Figure S7;** **Table 1** **; Table S3**). Like the TAP data, ciliopathy proteins were not enriched in SILAC datasets (**Table S3**). Thus, the SILAC data confirmed the IFT-B association found by TAP and increased the depth of our analysis.

We further validated the IFT-B associations in HEK293 kidney epithelial cells using co-immunoprecipitations followed by western blotting and showed that transiently transfected ARL13B-Flag immunoprecipitated IFT88 and IFT52 (**Figure 5C**). Finally, using dedicated yeast-two hybrid assays, human ARL13B was screened for direct interactions against a panel of 164 proteins, which contains most known IFT and ciliopathy proteins. Direct binary interactions of ARL13B were identified for IFT46 and IFT74 (**Figure 5D**).

In summary, we have identified the composition of epitope-tagged, cilium-localized ARL13B complexes and uncovered an association with IFT complex B via IFT46 and IFT74 interactions. Together with our *C. elegans* data, we conclude that IFT facilitates ciliary entry and/or retention of ARL13B/ARL-13 via direct interactions with the IFT-B complex.

## Discussion

To investigate mechanisms underpinning protein sequestration to ciliary membranes, we assessed how Joubert syndrome-associated ARL13B/ARL-13 is targeted to and restricted at ciliary membranes. We show that ARL13B/ARL-13 is compartmentalised within an evolutionarily conserved Inversin-like ciliary membrane subdomain and requires palymitoylation modification and RVVP motifs to prevent inappropriate targeting of *C. elegans* ARL-13 to the nucleus and distal ciliary regions. We also uncovered differential requirements for TZ and IFT genes in preventing ARL-13 accumulation at TZ and periciliary membranes (PCM). Mechanistically, MKS and NPHP genes, as well as DYF-13/TTC26, appear to regulate a TZ barrier to ARL-13 diffusion, whereas most examined IFT proteins regulate ARL-13 ciliary entry and/or retention via active transport processes. Consistent with this conclusion, human ARL13B interacts biochemically with the IFT-B complex via IFT46 and IFT74 interactions, and *C. elegans* ARL-13 can be observed to undergo IFT-like motility.

### Cell subtype- and age-dependent variation in the ARL13B/ARL-13 subciliary domain

Our localisation studies in oviduct and tracheal epithelial cells show that mammalian ARL13B joins a group of other ciliopathy proteins (Inversin/NPHP2, NPHP3 and NPHP9/NEK8) that localise to proximal ciliary compartments, excluding the TZ [4], [55], thus extending our previous finding for proximal ciliary targeting of *C. elegans* ARL-13 [35]. However, these proximal ciliary compartments are not universal because in various motile and non-motile ciliary sub-types, the ARL13B domain extends to the ciliary tips [18], [32], [34]. Also, ARL13B is not restricted to the same proximal compartment in renal epithelial cells as NPHP2, NPHP3 and NPHP9 (personal communication in [35]). An additional layer of compartment diversity stems from our finding that *C. elegans* ARL-13 extends to the ciliary tips of young larval cilia, before restricting to a proximal domain. Thus, the ARL13B/ARL-13 domain is differentially defined in different cell types and at different developmental stages, reflecting age and cell subtype-specific requirements for this G-protein. Another interesting age distinction is that we have only observed IFT-like motility for ARL-13 in young larval worms and not in later larvae or adults. Although there may be technical considerations that prevent us seeing ARL-13 processive movement in older worms (e.g., higher levels of diffusing signals obscuring IFT movements), our data indicates that as the cilium ages, the proportion of ARL-13 undergoing active transport may reduce compared to the fraction undergoing diffusion. Thus, for ciliary membrane proteins considered as potential IFT cargo, it may be fruitful to perform experiments on developing or newly formed cilia.

### MKS/NPHP modules and DYF-13 regulate the ARL-13 diffusion barrier at the TZ

Our work showing that ARL-13 readily diffuses at the middle segment membrane but fails to enter the adjacent TZ membrane subdomain clearly demonstrates an ARL-13 diffusion barrier at the *C. elegans* TZ. Using subcellular localisation and *in vivo* FRAP assays we were then able to show that this barrier is bidirectional and dependent on MKS and NPHP genes, but not most IFT genes. These observations are consistent with and extend published findings implicating a membrane diffusion barrier at the ciliary base, including a previous report by us and others showing that plasma membrane-associated RPI-2 (retinitis pigmentosa gene 2 orthologue) and transmembrane TRAM-1 (Sec61 ER translocon component) abnormally leak into the ciliary axonemes of TZ gene-disrupted worms [16]–[19], [21]. Indeed, our development of the first *in vivo* FRAP assay to measure barrier integrity and ciliary/periciliary exchange kinetics will help further dissection of ciliary ‘gating’ at the TZ.

Not all MKS, NPHP and IFT genes neatly fit our model, however. For example, the ARL-13 barrier appears mostly intact in TZ-associated *nphp-4* single mutants, despite previous findings that non-ciliary plasma transmembrane and membrane-associated proteins (RPI-2, TRAM-1) abnormally leak into the cilia of these worms [19]. Thus, NPHP-4 possesses selective ‘gating’ functions, required to prevent RPI-2 entry into cilia but not ARL-13 exit from cilia. In contrast, MKS-5 facilitates both these functions, indicating a more global function in TZ barrier regulation. Another example is *dyf-13/TTC26*, which is genetically and biochemically associated with the IFT-B complex [45], [56], [57]. Unlike other IFT mutants we tested, the TZ barrier is moderately disrupted in *dyf-13* single mutants, and even further compromised in *dyf-13;nphp-4* double mutants, suggesting a synthetic functional relationship between these genes. In *C. elegans*, DYF-13 has been placed in a distinct OSM-3/KIF17 accessory motor module with DYF-1/IFT70, on the basis that it is required for building at least part of the ciliary distal segment [45]. Surprisingly, although DYF-13 undergoes IFT [58], it is not yet known if this protein is required for IFT; thus it is possible that DYF-13 is peripherally associated with IFT complexes as a TZ-interacting cargo element, rather than a core component of the IFT machinery. Consistent with this notion, mammalian TTC26 is reported to be enriched at the TZ of mammalian photoreceptor and IMCD3 cells [59]. Future efforts focussing on the requirement of *dyf-13* for IFT and the integrity of the TZ in *dyf-13* and *dyf-13;nphp-4* mutants should be revealing.

How MKS, NPHP and DYF-13/TTC26 define the ARL-13 membrane diffusion barrier at the TZ is unknown. The absence of TZ proteins from our biochemically defined ARL13B complexes makes it unlikely that the barrier involves direct inhibitory interactions between ARL13B and these proteins; however, we cannot discount weak or transient interactions, nor technical limitations with standard TAP in identifying interactions with TZ-associated proteins, most of which are membrane proteins. More likely is that MKS and NPHP modules regulate TZ membrane lipid compositions or steric properties. Indeed, the latter is at the heart of the ‘picket fence’ membrane diffusion barrier model, where cytoskeletal-anchored membrane proteins form an obstacle barrier to molecular diffusion [60]. Consistent with this notion, the TZ contains unusual membrane-associated ultrastructural features such as Y-link connectors, the ciliary necklace and ciliary bracelet, all of which may contribute to a highly restricted and compacted TZ membrane that blocks free diffusion. Further support to this model comes from studies in worms and algae showing that disruption of MKS, MKS and JS genes cause Y-link loss [16], [19], [20].

However, TZ ultrastructural features are not found elsewhere in the axoneme and cannot explain the diffusion barrier preventing *C. elegans* ARL-13 entry into distal segment membranes. Nonetheless, there is evidence in our data that this barrier may be partially dependent on MKS, NPHP and *dyf-13* genes, as well as BBS genes, because in the corresponding mutants we found a significant number of worms with weak ARL-13 signals in more distal ciliary regions (**Figure S3B**). Also, our finding of a correlation between distal segment localisation of an ARL-13(ΔRVVP) variant together with reduced ciliary levels of CHE-13/IFT57 is consistent with a possible role for IFT in restricting ARL-13 distal segment entry. The availability of hypomorphic IFT mutants retaining distal segments would help to further address this issue.

### Role of IFT in active transport of ARL-13 and regulation of ARL-13 intraciliary mobility

**Figure 6:**
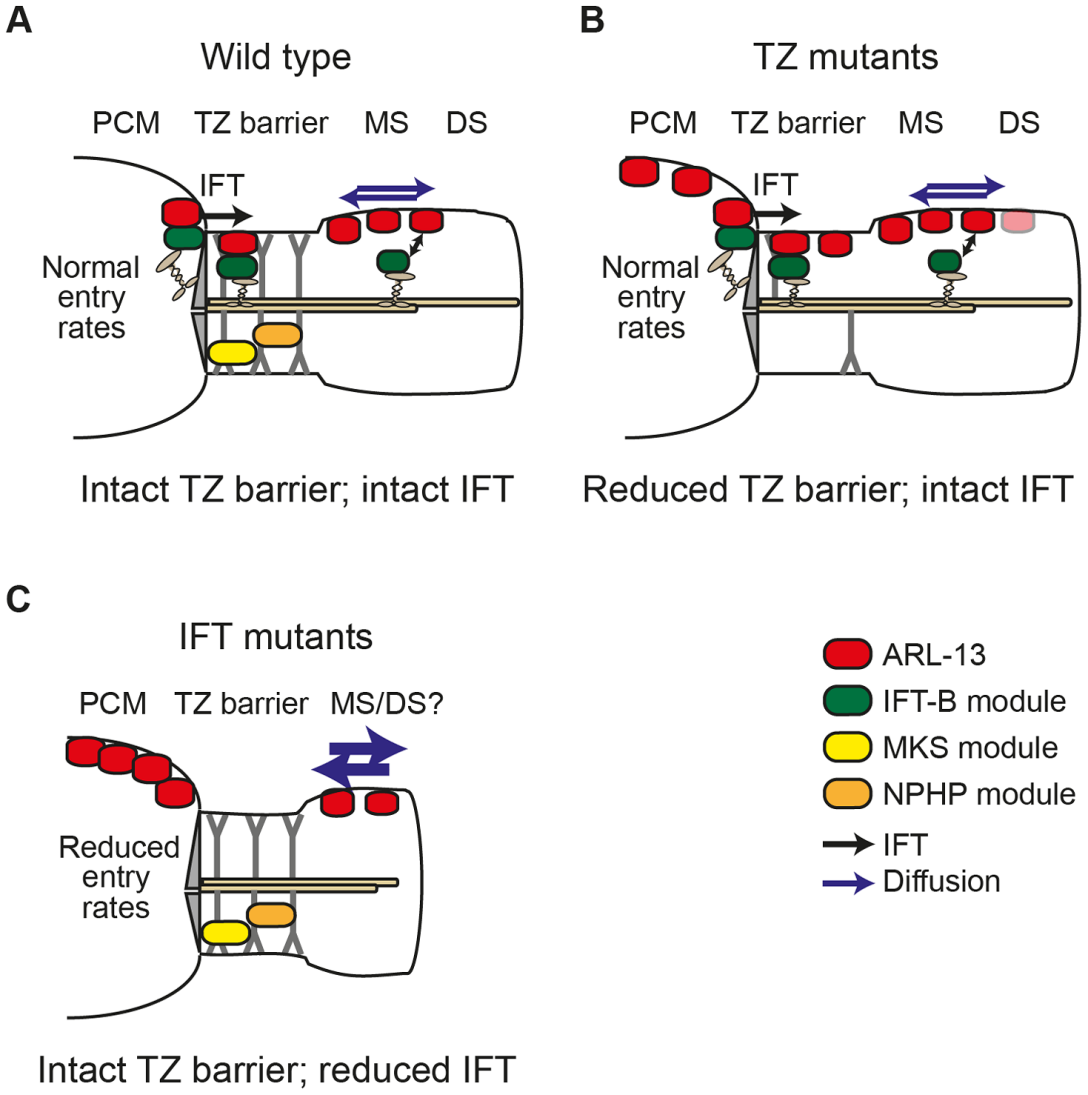
Model of differential roles for IFT and transition zone modules in defining the *C. elegans* ARL-13 ciliary membrane subdomain. (**A**) In wild type cilia, MKS and NPHP modules ensure an intact transition zone (TZ) barrier to ARL-13 diffusion (blue arrows), preventing ARL-13 exchange between the periciliary membrane (PCM) and the middle segment (MS) membrane. Through interactions with the IFT-B complex, IFT facilitates ARL-13 ciliary restriction by trafficking PCM-localised ARL-13 (derived from upstream transport and/or leakage out of the cilium) across the TZ barrier into the MS. Alternatively, IFT trains in the MS could prevent ARL-13 from exiting cilia (double arrow). (**B**) In TZ module gene mutants, the TZ barrier is weakened (e.g., via loss of Y-link structures) and ARL-13 readily exchanges between PCM and MS membranes. Although IFT remains intact [19], it cannot fully compensate for the weakened barrier, thus a steady state pool of ARL-13 is observed at TZ and PCM membranes. Low level ARL-13 signals (pink shade) are found in distal segment (DS) regions, suggesting a partial defect in the diffusion barrier at the MS/DS boundary. (**C**) In IFT module gene mutants, the TZ barrier to ARL-13 diffusion is mostly intact; however, loss of IFT causes reduced entry of ARL-13 into cilia (or increased exit from cilia), resulting in PCM accumulation of ARL-13. IFT mutants also display increased ARL-13 diffusion kinetics (thicker blue arrows) in the cilium.

A number of pieces of evidence from this study support our conclusion that IFT restricts ARL-13 to its compartment via active transport mechanisms versus more passive processes such as regulation of a TZ diffusion barrier. First, ARL-13 undergoes IFT-like motility. Second, the TZ diffusion barrier appears mostly intact in most IFT gene mutants (except *dyf-13*, discussed above). Third, human ARL13B interacts directly with IFT-B complexes via IFT46/74 interactions. The very specific accumulation of ARL-13 at the PCM of IFT mutants and not elsewhere in the cell suggests IFT facilitates ARL-13 trafficking from the PCM into cilia and/or prevents ARL-13 from exiting cilia and accumulating at the PCM. Although we have been unable to distinguish between these two non-mutually exclusive possibilities, our biochemical and IFT motility data suggests that the mechanism involves ARL-13 directly interacting with IFT trains as cargo. In the first scenario, PCM-localised ARL-13, derived from upstream transport or leakage out of the cilium, would be captured by IFT-B complexes at the basal body and then moved back into the cilium, across the TZ, by anterograde IFT. In the alternative scenario, ARL13B could be held in cilia by dynamic association with moving IFT trains, perhaps with rapid on/off rates. In both cases, IFT disruption would lead to PCM accumulation of ARL-13. Thus, as shown in our model (**Figure 6**), IFT functions in a distinct manner to TZ-associated modules in restricting ARL-13 to a subciliary membrane compartment. Whilst TZ modules establish the TZ barrier to ARL-13 diffusion, IFT modules actively transport ARL-13 across the barrier or retain it in cilia.

Since the ARL-13 TZ diffusion barrier does not depend on IFT genes, we were surprised that ARL-13 diffusion rates were increased at the middle segment membrane of IFT mutants, suggesting that IFT restricts ARL-13 mobility in cilia. One possible explanation is that ARL-13 diffusion rates are retarded due to frequent interactions of ARL-13 with IFT trains as they move through the ARL-13 compartment. Alternatively, through its role in targeting proteins to cilia, IFT may regulate steric hindrance to free diffusion at the ciliary membrane. In *Chlamydomonas*, IFT regulates the mobility of transmembrane PKD2; however, unlike the increased FRAP rates we find for ARL-13 in IFT mutant worms, CrPKD2 displays reduced FRAP rates in *fla-10^ts^* (kinesin-II) algae [9]. Another difference is that PKD2 ciliary concentrations are elevated in IFT-disrupted algae, worms and mice [9], [29], [30], whereas this is not the case for ARL-13 in *C. elegans* IFT mutants (this study; data not shown). Thus, IFT appears to differentially regulate the ciliary transport and mobility of PKD2 and ARL-13. Future efforts using FRAP or photoconversion techniques will help to further tease out the role of IFT in regulating protein dynamics at the ciliary membrane.

### Affinity proteomics identifies new avenues for investigating ARL-13 transport and function

Using affinity proteomics, we identified reproducible high ranking associations between ARL13B and most of the proteins that form a ∼500 kDa IFT-B core in *Chlamydomonas* [52]. The absence of IFT-A, kinesin-2, IFT-dynein and BBSome proteins indicates a specific interaction between ARL13B and IFT-B complexes, which we mapped to IFT46 and IFT74 using yeast-two-hybrid analyses. These findings indicate that ARL13B physically interacts with IFT complex B, which as discussed above may explain how IFT controls ARL-13 retention and mobility at ciliary membranes. Furthermore, the IFT-B interaction is consistent with a reciprocal role for ARL-13 in regulating anterograde IFT, previously reported by us and others [35], [36]. Interestingly, GDP-locked ARL13B, as well as the R79Q and R200C ARL13B patient variants, retained high affinity binding of the IFT-B complex, indicating that ARL13B/IFT-B associations do not require GDP/GTP exchange or R79/R200 residues. Our affinity proteomics data also indicate that wild type ARL13B complexes contain karyopherin-beta proteins involved in nucleocytoplasmic transport. Since ARL-13/ARL13B is almost exclusively localised in cilia [32]–[35], the karyopherin associations are likely occurring within cilia or en route to the cilium. Indeed, nucleocytoplasmic transport machinery (Ran, nuclear pore complex subunits and karyopherin beta/importin proteins) localise at the primary cilium of cultured mammalian cells, and are required for entry of proteins into cilia, including KIF17 [23]–[27]. Although more work needs to be performed, it is tempting to speculate that ciliary retention of ARL13B requires karyopherin-beta protein function; alternatively ARL13B could be a regulator of nucleocytoplasmic transport proteins operating in the cilium. Other interesting proteins with high affinity binding within ARL13B complexes are proteins associated with cullin-RING E3 ubiquitin ligase (CRL) complexes, namely DCAF8 (DDB1 and CUL4 associated factor) and CAND1/CAND2 (cullin-associated and neddylation-dissociated). DCAFs are thought to facilitate the recruitment of substrates onto CRL4 scaffolds and CAND1/2 proteins are known to negatively regulate the E3 ligase activity of CRLs (reviewed in [61]). Interestingly, it was recently shown that the small ubiquitin-like modifier (SUMO) is conjugated to, and functionally regulates, ARL-13/ARL13B [40]. Although speculative, our data could suggest that ARL13B is targeted for CRL4/DCAF8-mediated ubiquitination. Alternatively, and consistent with the CAND1/2 associations, ARL13B may regulate CRL-mediated processes.

### Concluding remarks

We have performed a thorough analysis of transport mechanisms organising the ARL-13/ARL13B ciliary signalling subdomain. Our findings reveal differential requirements for sequence motifs, IFT-related complexes and TZ-associated ciliopathy modules in defining an ARL-13 subciliary membrane domain. We find that MKS and NPHP modules, as well as DYF-13/TTC26, regulate a TZ barrier to ARL-13 diffusion, whereas most IFT proteins facilitate ARL-13 ciliary entry and/or retention predominantly via active transport mechanisms. Furthermore, our protein complex and protein-protein interaction data represents a good starting point to further address mechanisms of ARL-13 ciliary protein transport and function, and provides new research avenues for investigating the pathomechanisms of Joubert Syndrome-related disorders.

## Materials and Methods

### *C. elegans* strains, alleles and transgenes

C. elegans worms were maintained and cultured at 20°C using standard techniques [62]. Strains employed were *N2 (Bristol), N2;oqEx58[Parl-13::ARL-13::GFP+pRF4]*, *N2;oqEx[Parl-13::ARL-13::tdTomato+pRF4]*, *N2;mnIs17[Posm-6::OSM-6::gfp]*, *N2;oqEx51[Parl-13::ARL-13(rPal)::GFP+pRF4]*, *N2;oqEx[Parl-13::ARL-13(Δ285–370)::GFP+pRF4]*, *N2;oqEx[Parl-13::ARL-13(T38N)::GFP+pRF4]*, *N2;oqEx[Parl-13::SYN-16::DsRed]*, *N2;myEx[Pche-12::CHE-13*::*mCherry*+*Punc*-*122::GFP]*, *N2;ejEx[Pkap-1::KAP-1::GFP+pRF4]*, MX349: *dpy-5(e907); nxEx[Pmksr-1::MKSR*-*1*::*GFP+dpy-5 (+)]*, *che-2(e1033)*, *dyf-3(m185)*, *che-13(e1805)*, *dyf-6(m175)*, *dyf-13(mn396)*, *che-11(e1810)*, *dyf-2(m160)*, *xbx-1(ok279)*, *che-3(e1124)*, *klp-11(tm324)*, *osm-3(p802)*, *mks-5(tm3100)*, *mksr-1(ok2092)*, *mksr-2(tm2452)*, *mks-2(nx111)*, *nphp-4(tm925)*, *bbs-8(nx77)*, *bbs-7(n1606)*, *bbs-1(ok1111)*, *unc-59(e261)*, *unc-61(n3169)*.

### Genetic crossing

Standard genetic crossing techniques were used to make double mutants and to introduce transgenes into genetic backgrounds. PCR using primers flanking deletions were used to follow *nx77*, *tm324*, *ok2092*, *nx111*, *tm925* and *ok990* mutations. All other mutations were followed using a dye-filling assay or an Unc phenotype (*unc-59/61*).

### Generation of fluorescence tagged *C. elegans arl-13* constructs

All constructs were generated by fusion PCR as previously described [63]. For *arl-13p::arl-13::tdTomato* and *arl-13p::arl-13(Δ285–370)::gfp*, plasmid amplified *gfp* (from pPD95.77) or tdTomato (from pPD95.75/Pwrt-2::CDC-6::tdTomato; gift of E. Kipreos; University of Georgia, USA) was fused in frame with genomic DNA fragments containing 300 bp of *arl-13* 5′ untranslated region, and either the entire exonic/intronic *arl-13* sequence (1–3298 nucleotides; not including the stop codon) for the tdTomato construct or a truncated sequence (1–2998 nucleotides) for the amino acid 285–370 deletion construct. For the *arl-13p::arl-13(T38N)::gfp* variant, a PCR fragment was genomically amplified to contain the *arl-13* promoter (214 bp) plus the first two exons of *arl-13* to nucleotide position 1001, with nucleotides 999–1001 (threonine codon) altered by primer design to create an asparagine codon. This fragment was then fused to a PCR fragment containing the remainder of *arl-13* sequence (minus the stop codon), and the resultant amplicon was fused in frame with *gfp*. All constructs were coinjected at 1–10 ng/µl with 50 ng/µl pRF4 into N2 worms to generate roller transgenic animals harboring extrachromosomal arrays.

### Fluorescence recovery after photobleaching

Early adult worms were immobilised with 0.1 µm polystyrene microspheres (Polysciences) on a 10% agarose pad and covered with a coverslip. Experiments were performed on a Nikon Eclipse Ti microscope fitted with a 100×1.4NA Plan APO VC objective (Nikon), a 50 mW 488 nm laser, and CSU-X1 spinning disk unit (Yokogawa). Samples were excited using the 488 nm laser at 50% and images were recorded using a charge-coupled device camera (iXon EM-CCD, Andor Technology) controlled by Andor Technology iQ 2.6 software. Samples were imaged pre-bleach, and then bleached using a single pulse of the 488 nm laser at 100% with a dwell time of 100 µs. Images were recorded immediately post-bleach, at 15 s, 30 s, 60 s, 120 s, 180 s, 240 s, 360 s, 480 s, and 600 s for intraciliary FRAP experiments, and post-bleach at 15 s, 30 s, 60 s, 120 s, 180 s, 240 s, 300 s, 600 s, 900 s, and 1200 s for periciliary membrane (PCM) and cilium compartment FRAP experiments. For intraciliary FRAP experiments EM gain was set to 6; for compartment FRAP experiments the EM gain was set to 20, with an exposure time of 50 ms in all experiments. Images were imported into ImageJ and converted into a stack. Photobleached and non-photobleached regions of the cilium were selected and intensity measured at each timepoint. After background subtraction, ratios of bleached∶non-bleached regions were calculated. Ratios were normalised to pre-bleach ratio. Curves were fitted and half-time recovery calculated using GraphPad Prism 5.0 software. Most FRAP curves (saturation plots) returned high goodness-of-fit statistics (R-squared>0.75), except for *xbx-1* (ciliary, PCM, and total FRAP), *dyf-6* (ciliary and total FRAP), *che-2, dyf-13;nphp-4* (total FRAP), and *mks-5* (total FRAP). These exceptions correlate with experiments where FRAP recovery kinetics and levels were low, which makes it more difficult to get a good fit.

### Cell culture

HEK293T cells were cultured as described previously [64]. For SILAC experiments, HEK293T cells were grown in SILAC DMEM (PAA) supplemented with 3 mM L-Glutamine (PAA), 10% dialysed fetal bovine serum (PAA), 0.55 mM lysine and 0.4 mM arginine. Light SILAC medium was supplemented with ^12^C_6_, ^14^N_2_ lysine and ^12^C_6_, ^14^N_4_ arginine. Heavy SILAC medium was supplemented with either ^13^C_6_ lysine and ^13^C_6_, ^15^N_4_ arginine or ^13^C_6_, ^15^N_2_ lysine and ^13^C_6_, ^15^N_4_ arginine. 0.5 mM proline was added to all SILAC media to prevent arginine to proline conversion [65]. All amino acids were purchased from Silantes. For DNA transfections, HEK293T cells were seeded, grown overnight, and then transfected using PEI transfection.

### Affinity purification of protein complexes

Protein complex detection and comparison was done essentially as described before [54]. For one step Strep purifications, SF-TAP tagged proteins and associated protein complexes were purified essentially as described earlier [50]. HEK293T cells, transiently expressing the SF-TAP tagged constructs were lysed in lysis buffer containing 0.5% Nonidet-P40, protease inhibitor cocktail (Roche) and phosphatase inhibitor cocktails II and III (Sigma-Aldrich) in TBS (30 mM Tris-HCl (pH 7.4), 150 mM NaCl) for 20 minutes at 4°C. After sedimentation of nuclei at 10,000× g for 10 minutes, the protein concentration of the cleared lysates was determined by Bradford before equal protein amounts were transferred to Strep-Tactin-Superflow beads (IBA) and incubated for one hour before the resin was washed three times with wash buffer (TBS containing 0.1% NP-40, phosphatase inhibitor cocktail II and III). The protein complexes were eluted by incubation for 10 minutes in Strep-elution buffer (IBA). The eluted samples were combined before concentration using 10 kDa cut-off VivaSpin 500 centrifugal devices (Sartorius Stedim Biotech) and pre-fractionation using SDS-Page and in-gel tryptic cleavage as described elsewhere [64]. For SF-TAP analysis, the constructs were expressed and cells harvested as described above. The cleared supernatant was incubated for one hour at 4°C with Strep-Tactin superflow (IBA). Subsequently, the resin was washed three times in wash buffer. Protein baits were eluted with Strep-elution buffer. For the second purification step, eluates were transferred to anti-Flag M2 agarose (Sigma-Aldrich) and incubated for one hour at 4°C. Beads were washed three times with wash buffer and proteins eluted with Flag peptide (200 µg/ml, Sigma-Aldrich) in TBS. After purification, samples were precipitated with chloroform and methanol and subjected to in-solution tryptic cleavage as described before [64].

### Mass spectrometry and data analysis

LC-MS/MS analysis was performed on an Ultimate3000 RSLCnano HPLC system (Thermo Fisher Scientific) coupled to a LTQ Orbitrap Velos mass spectrometer (Thermo Fisher Scientific) by a nano spray ion source. Tryptic peptide mixtures were automatically injected and loaded at flow rate of 6 µl/min in 0.5% trifluoroacetic acid in HPLC grade water onto a nano trap column (100 µm i.d. ×2 cm, packed with Acclaim PepMap RSLC C18, 3 µm, 100 Å, Thermo Fisher Scientific). After 5 minutes, peptides were eluted and separated on the analytical column (Acclaim PepMap RSLC C18, 2 µm, 100 Å, 75 µm i.d. ×25 cm, nanoViper, Thermo Fisher Scientific) by a linear gradient from 5% to 35% of buffer B (80% acetonitrile, 0.08% formic acid) in buffer A (2% acetonitrile, 0.1% formic acid in HPLC grade water) at a flow rate of 300 nl/min over 80 minutes. Remaining peptides were eluted by a short gradient from 40% to 100% buffer B in 5 minutes. Eluted peptides were analyzed by the LTQ Orbitrap Velos mass spectrometer. From the high resolution MS pre-scan with a mass range of 300 to 1500, the ten most intense peptide ions were selected for fragment analysis in the linear ion trap if they exceeded an intensity of at least 250 counts and if they were at least doubly charged. The normalized collision energy for CID was set to a value of 35 and the resulting fragments were detected with normal resolution in the linear ion trap. The lock mass option was activated, the background signal with a mass of 445.12002 was used as lock mass [66]. Every ion selected for fragmentation was excluded for 20 seconds by dynamic exclusion. For SILAC experiments, all acquired spectra were processed and analyzed using the MaxQuant software [67] (version 1.3.0.5) and the human specific Uniprot database (Version 11/07/2012). Cysteine carbamidomethylation was selected as fixed modification and methionine oxidation and protein acetylation was allowed as variable modifications. The peptide and protein false discovery rates were set to 1%. Contaminants like keratins were removed. Proteins identified and quantified by at least two peptides per experiment in at least two of three independent experiments were considered for further analysis. Because hundreds of proteins were enriched with ARL13B, we had to set a ratio threshold instead of using significance values determined by MaxQuant. Only proteins enriched at least 2-fold were considered for further analysis. For these proteins the significance A was determined for the comparison of wild type ARL13B to the mutant forms.

For non-quantitative experiments, the raw data were analyzed using Mascot (Version 2.4) and Scaffold (Proteome Software) against the human subset of the SwissProt Database (Version 18/05/2012, 536029 sequences) as earlier described [64]. Proteins were considered to be specific protein complex components if they were identified in at least two of three experiments with two or more peptides (peptide probability >80%). The protein probability threshold was set to 95%. Likely false positives were removed if they were identified in 15% or more non-ARL13B SF-TAP experiments.

### Dedicated yeast two-hybrid interaction assay

The direct interaction between ARL13B and other ciliary proteins was tested using a GAL4-based yeast two-hybrid system (Hybrizap, Stratagene, USA). The DNA binding domain (GAL4-BD) fused to full length ARL13B was used as a bait to test the interaction with previously described ciliopathy and cilium-associated proteins fused to an activation domain (GAL4-AD). Constructs encoding GAL4-BD and GAL4-AD fusion proteins were co-transformed in yeast strain PJ69-4A. The direct interaction between baits and preys induced the activation of the reporter genes, resulting in the growth of yeast colonies on selective media (deficient of histidine and adenine) and induction of α-galactosidase and β-galactosidase colorimetric reactions [68].

### Immunohistochemistry of mouse tissues

Following perfusion with 4% paraformaldehyde (PFA)/PBS, mouse tissues were dissected out and incubated in 4% PFA/PBS at 4°C overnight. Fixed tissues were transferred to 30% sucrose/PBS solution, incubated for a few days, and embedded in OCT compound and stored at −80°C before use. Frozen tissue sections immunostained using anti-ARL13B [33] and anti-acetylated tubulin antibodies.

### ARL13B-Flag co-immunoprecipitation assay

HEK293T cells were transfected with a C-terminal Flag-tag expression vector with or without (as “Mock”) human full-length Arl13b cDNA. Two days after transfection, the cell lysates were prepared using extraction buffer (50 mM Tris-HCl (pH 7.5), 100 mM NaCl, 1% NP-40, 5 mM MgCl2, 1 mM DTT, 0.1 mM PMSF, 2 µg/ml aprotinin, 2 µg/ml leupeptin) and subjected to immunoprecipitation using anti-Flag M2 beads (Sigma). Immunocomplexes were then subjected to Western blot analysis using anti-IFT88 antibody (Proteintech), anti-IFT52 antibody (Proteintech) and anti-INVS antibody (a generous gift from D. Shiba and T. Yokoyama), and anti-Flag antibody (Sigma).

## Supporting Information

Figure S1 Developmental timecourse of ARL-13 compartment formation and mobility of ARL-13 at the ciliary membrane, linked to Figure 1. (**A**) Phasmid cilia from wild-type worms at different larval and adult stages expressing the indicated transgene-encoded protein. Arrowhead; basal body. Bar; 1 µm. (**B**) Phasmid cilia of worms expressing ARL-13::GFP showing FRAP recovery after photobleaching ∼40% of proximal ciliary signal. Bar; 1 µm.
(PDF)

Figure S2 Analysis of ARL-13 sequence variant localisation and function, linked to Figure 2. (**A**) Amphid neuronal cell bodies from wild-type worms expressing the TGN marker SYN-16::dsRed with either ARL-13(Δ203–370)::GFP or ARL-13(ΔRVVP)::GFP. n; nucleus. Bar; 1 µm. (**B, C**) Dye-fill images of *arl-13(tm2322)* and wild-type worms expressing the indicating ARL-13 sequence variant. Bars; 5 µm. (**D**) Amphid images of worms expressing CHE-13/IFT57::mCherry and the indicated ARL-13 sequence variant. BB; basal body regions. ax; axonemes. Bar; 2 µm.
(PDF)

Figure S3 Quantification of ARL-13 signals at the periciliary membrane, cell body and transition zone in ciliopathy and ciliogenic gene mutants, linked to Figure 3. (**A**) Percentage worms exhibiting ARL-13::GFP accumulation at the periciliary membrane of phasmid neurons in the indicated mutant genotype. (**B**) Percentage worms of the indicated genotype displaying punctate ARL-13::GFP signals in the cell bodies of phasmid neurons. Images are of phasmid neurons showing ARL-13::GFP signals in the cell bodies of *mks-2;nphp-4* mutants. TZ; transition zone. PCM; periciliary membrane. Bars; 2 µm. (**C**) Assessment of ARL-13::GFP localisation at the TZ of phasmid neurons. TZ localisations typically identifiable as a narrowing of ARL-13 signal between the ciliary axonemal and wider periciliary membrane compartment.
(PDF)

Figure S4 Additional ARL-13 FRAP curves (bleaches of PCM or cilium) and images, linked to Figure 4. (**A**) Each graph shows the PCM and ciliary FRAP curves. All curves are subtracted for background photobleaching and any very low level recovery from PCM+ ciliary bleaches (shown in Figure 4). Data shows that bleaching of one pool (PCM or cilium) correlates with a signal reduction in the other pool; thus, recoveries come from the non-bleached pool (PCM or cilium). (**B**) Representative images from phasmid cilia photobleaching experiments. Brackets denote bleached regions. All images identically imaged and scaled. Bars; 2 µm.
(PDF)

Figure S5 Additional ARL-13 FRAP curves (bleaches of PCM+cilium) and quantitative analysis of bleached region in partial ciliary FRAP experiments, linked to Figure 4. (**A**) Representative FRAP images after photobleaching entire PCM+ciliary ARL-13 signals in phasmid neurons. Bar; 1 µm. (**B**) Representative FRAP images after photobleaching ∼40% of proximal-most ARL-13 signals in phasmid cilia. Bars; 1 µm. (**C**) Box and whisker (min to max) distribution plots showing the % area, length and intensity of photobleached ARL-13::GFP in the partial ciliary FRAP experiments shown in Figure 4C.
(PDF)

Figure S6 Strep/Flag (SF)-tagged ARL13B localises to hTERT-RPE1 primary cilia, linked to Figure 5. hTERT-RPE1 cells transiently transfected with N-terminally SF (Strep-Flag)-tagged ARL13B or C-terminally SF-tagged ARL13B show specific ciliary localisation of human ARL13B. Green; anti-Flag antibody staining. Red; anti-polyglutamylated tubulin antibody staining. Bars; 10 µm.
(PDF)

Figure S7 Investigation of ARL13B complexes using SILAC-based quantitative affinity proteomics, linked to Figure 5. Detection of proteins associated with wild type (WT) ARL13B protein complexes in HEK293 cells. Plotted are log2 ratios of proteins enriched in SF-ARL13B(WT) versus SF-control purifications (x-axis) and log2 intensities (y-axis) for each protein identified and quantified in at least two of four biological replicates. Enriched proteins (ratio>2) are plotted in green.
(PDF)

Movie S1 Time-lapse video recording (real time) of ARL-13::GFP processive movement along an amphid channel cilium of a wild-type worm (L1 stage) expressing ARL-13::GFP. Video captured at 3 frames per second.
(AVI)

Movie S2 Rotating Z-stacked and volume rendered images of PHA/B cilia in adult worms of the indicated genotype expressing ARL-13::GFP. In N2 and *che-3* images, ARL-13::GFP signals it the PQR cilium are also evident. TZ; transition zone. Asterisk; periciliary membrane accumulation. Bars; 2 µm.
(AVI)

Movie S3 Rotating Z-stacked image of entire PHA/B neurons showing ARL-13::GFP localisation in wild-type (N2) worms. Bar; 10 µm.
(AVI)

Movie S4 Rotating Z-stacked image of entire PHA/B neurons showing ARL-13::GFP localisation in a *che-2/IFT80* mutant. Bar; 10 µm.
(AVI)

Movie S5 Rotating Z-stacked image of entire PHA/B neurons showing ARL-13::GFP localisation in a *che-11/IFT140* mutant. Bar; 10 µm.
(AVI)

Movie S6 Rotating Z-stacked image of entire PHA/B neurons showing ARL-13::GFP localisation in a *che-3/DHC* mutant. Bar; 10 µm.
(AVI)

Movie S7 Rotating Z-stacked image of entire PHA/B neurons showing ARL-13::GFP localisation in an *osm-12/BBS7* mutant. Bar; 10 µm.
(AVI)

Movie S8 Rotating Z-stacked image of entire PHA/B neurons showing ARL-13::GFP localisation in an *mks-5/RPGRIP1L* mutant. Bar; 10 µm.
(AVI)

Movie S9 FRAP movies following bleaching of periciliary signals (denoted by brackets) in worms of the indicated genotype expressing ARL-13::GFP. All movies taken from phasmid cilia. Bars; 2 µm.
(AVI)

Movie S10 FRAP movies following bleaching of ciliary signals (denoted by brackets) in worms of the indicated genotype expressing ARL-13::GFP. All movies taken from phasmid cilia. Bars; 2 µm.
(AVI)

Movie S11 FRAP movies following bleaching of periciliary + ciliary signals in worms of the indicated genotype expressing ARL-13::GFP. All movies taken from phasmid cilia. Bars; 2 µm.
(AVI)

Movie S12 FRAP movies following bleaching of proximal or distal regions of ARL-13::GFP middle segment signals. Note the faster recovery in *dyf-6*/*IFT46* mutants vs the WT worms. Bars; 1.5 µm.
(AVI)

Table S1 Statistical analysis of periciliary/ciliary localisation data, linked to Figure 3. Shown are pairwise comparison of all combinations performed using 1-way ANOVA, Bonferroni corrected. ***P≤0.001. **P≤0.01. * P≤0.05. ns (not significant); P>0.05.
(XLSX)

Table S2 Human ARL13B complex proteins identified using tandem affinity proteomics, linked to Figure 5. Shown are peptide numbers (no.) and sequence (Seq) cover for proteins co-purified with SF (Strep/Flag)- tagged human ARL13B in HEK293 cells by SF-TAP. Specifically, the table shows data from 4 experiments using three different ARL13B constructs; SF-ARL13B(WT) (N-terminally tagged; 2 experiment average), ARL13B-SF(WT) (C-terminally tagged; 1 experiment) and SF-ARL13B(T35N) (predicted GDP-locked; 1 experiment). Final two columns show 4 experiment averages, and the list is sorted according to the average peptide number (highest to lowest).
(XLSX)

Table S3 Human ARL13B complex proteins identified using SILAC-based quantitative affinity proteomics, linked to Figure 5. **Columns D, E**; four experiment averages showing protein enrichment ratios and p-values from Strep/Flag (SF)-tagged ARL13B(WT) purifications quantified against purifications of the SF tag alone. **Columns F–I**; peptide enrichment scores from the 4 individual experiments. n.def. (not defined).
(XLSX)
