# Supplementary figures and images for: Active Transport and Diffusion Barriers Restrict Joubert Syndrome-Associated ARL13B/ARL-13 to an Inv-like Ciliary Membrane Subdomain
Source: PLoS Genet. 2013 Dec 5;9(12):e1003977. doi: 10.1371/journal.pgen.1003977 (PMC3854969; doi:10.1371/journal.pgen.1003977)

**A**

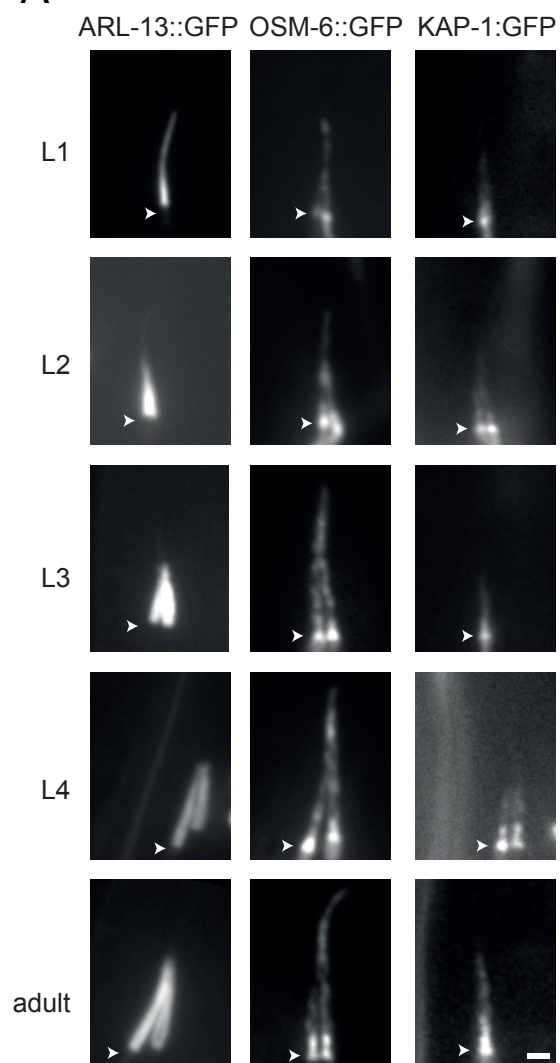

**B**

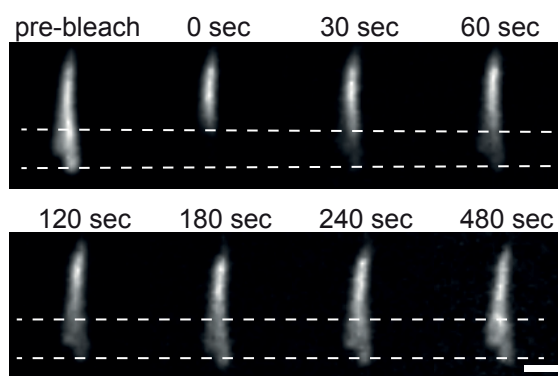

Supplement: Figure S1 — Developmental timecourse of ARL-13 compartment formation and mobility of ARL-13 at the ciliary membrane, linked to Figure 1. (A) Phasmid cilia from wild-type worms at different larval and adult stages expressing the indicated transgene-encoded protein. Arrowhead; basal body. Bar; 1 µm. (B) Phasmid cilia of worms expressing ARL-13::GFP showing FRAP recovery after photobleaching ∼40% of proximal ciliary signal. Bar; 1 µm. (PDF) [file pgen.1003977.s001.pdf]

A

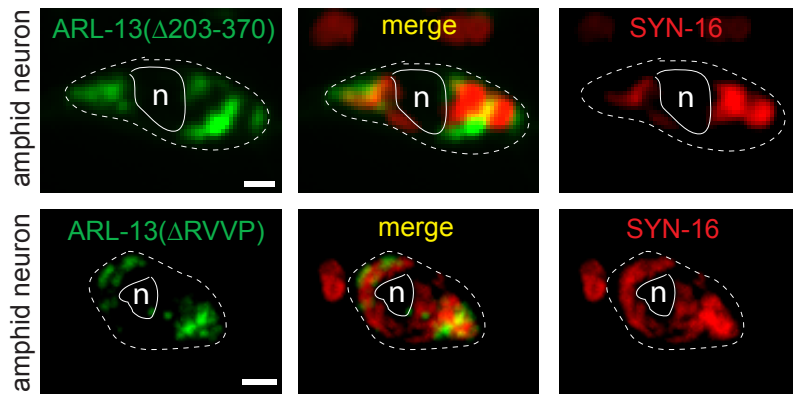

B

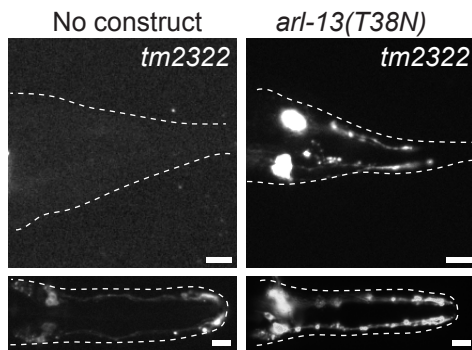

C

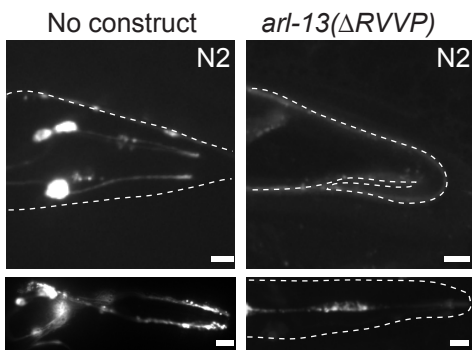

D

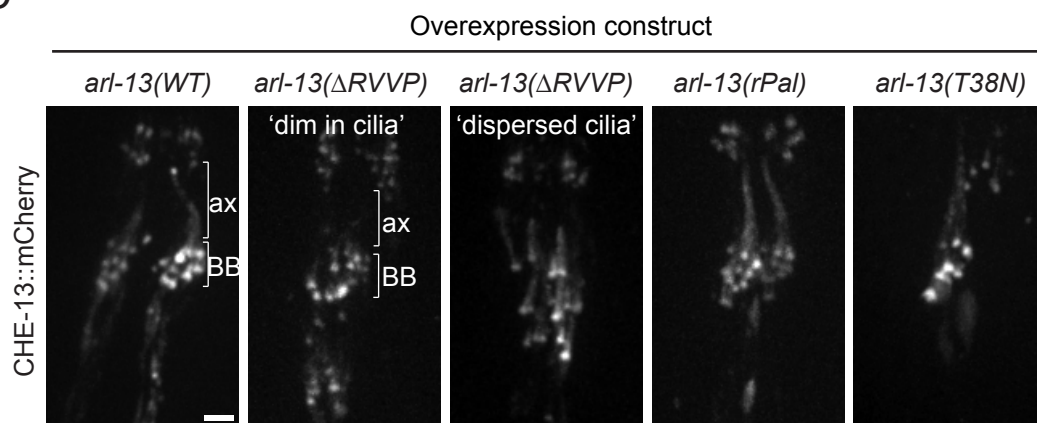

Supplement: Figure S2 — Analysis of ARL-13 sequence variant localisation and function, linked to Figure 2. (A) Amphid neuronal cell bodies from wild-type worms expressing the TGN marker SYN-16::dsRed with either ARL-13(Δ203–370)::GFP or ARL-13(ΔRVVP)::GFP. n; nucleus. Bar; 1 µm. (B, C) Dye-fill images of arl-13(tm2322) and wild-type worms expressing the indicating ARL-13 sequence variant. Bars; 5 µm. (D) Amphid images of worms expressing CHE-13/IFT57::mCherry and the indicated ARL-13 sequence variant. BB; basal body regions. ax; axonemes. Bar; 2 µm. (PDF) [file pgen.1003977.s002.pdf]

A

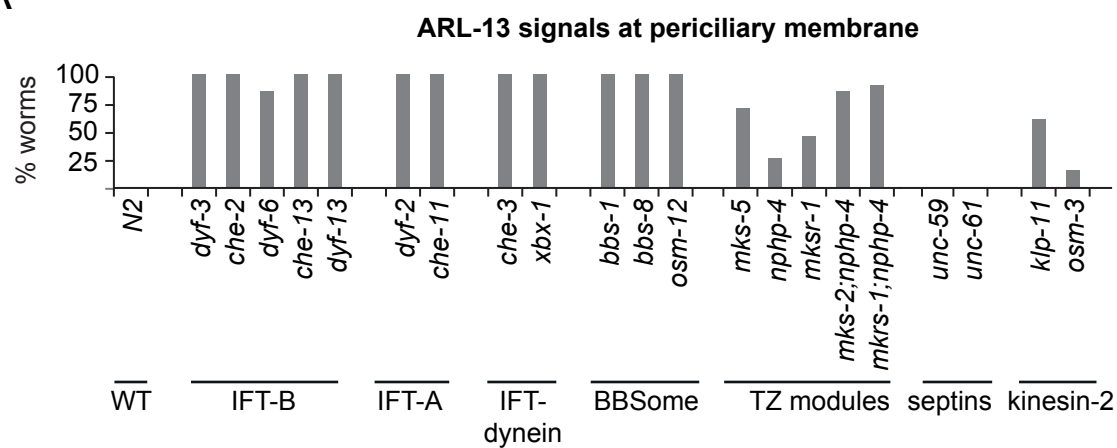

B

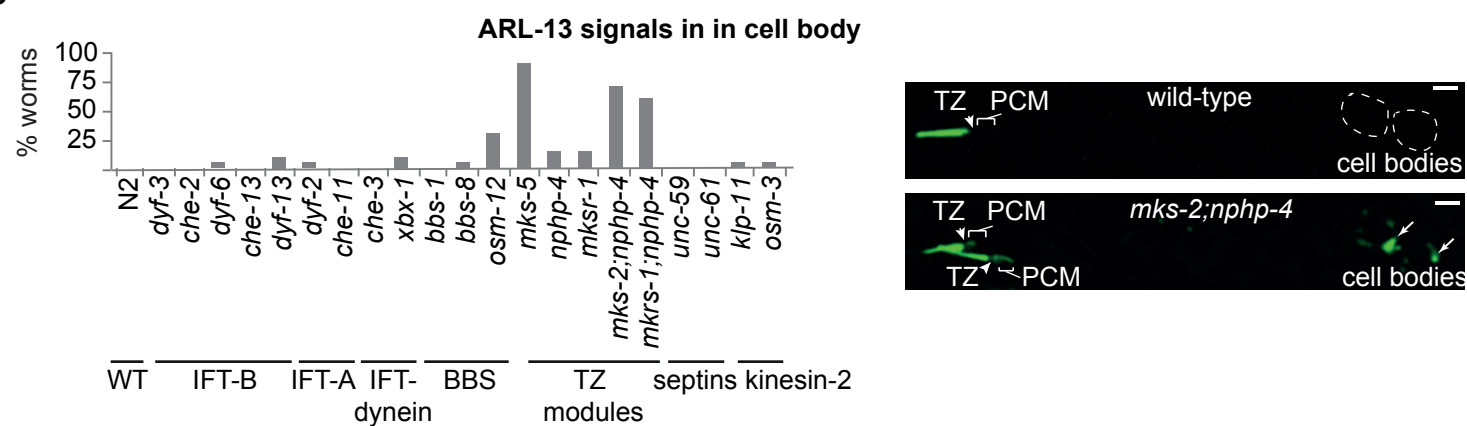

C

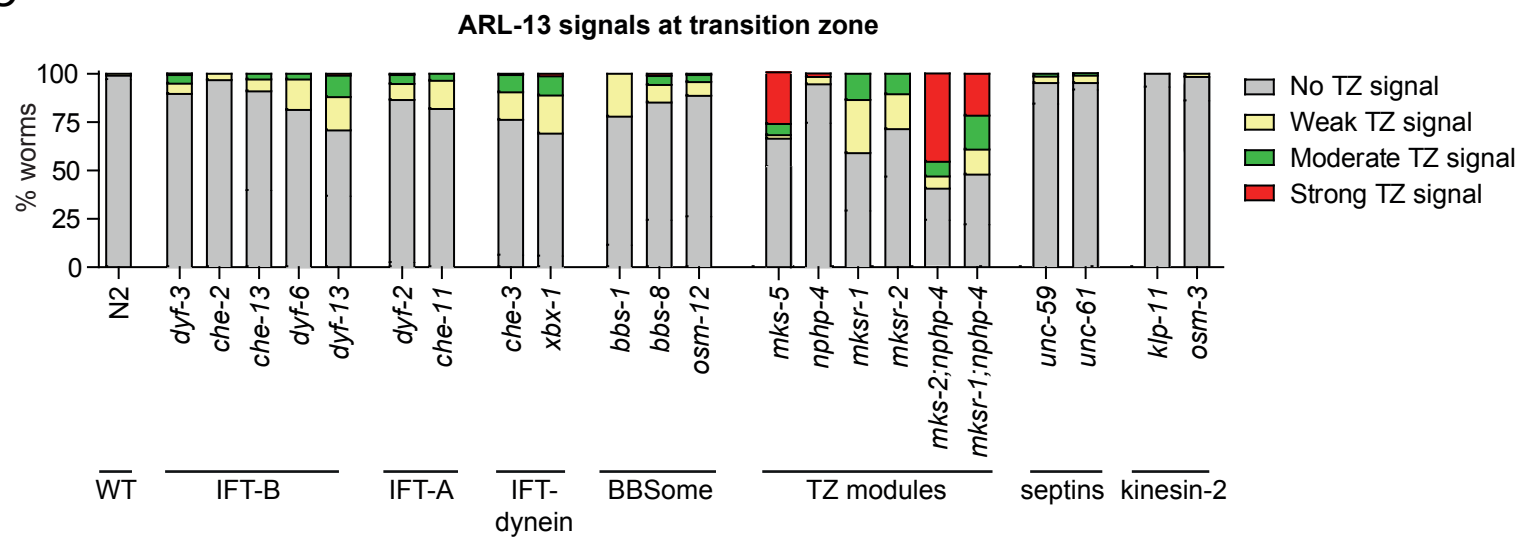

Supplement: Figure S3 — Quantification of ARL-13 signals at the periciliary membrane, cell body and transition zone in ciliopathy and ciliogenic gene mutants, linked to Figure 3. (A) Percentage worms exhibiting ARL-13::GFP accumulation at the periciliary membrane of phasmid neurons in the indicated mutant genotype. (B) Percentage worms of the indicated genotype displaying punctate ARL-13::GFP signals in the cell bodies of phasmid neurons. Images are of phasmid neurons showing ARL-13::GFP signals in the cell bodies of mks-2;nphp-4 mutants. TZ; transition zone. PCM; periciliary membrane. Bars; 2 µm. (C) Assessment of ARL-13::GFP localisation at the TZ of phasmid neurons. TZ localisations typically identifiable as a narrowing of ARL-13 signal between the ciliary axonemal and wider periciliary membrane compartment. (PDF) [file pgen.1003977.s003.pdf]

**A**

● PCM signal intensity      ■ cilium signal intensity

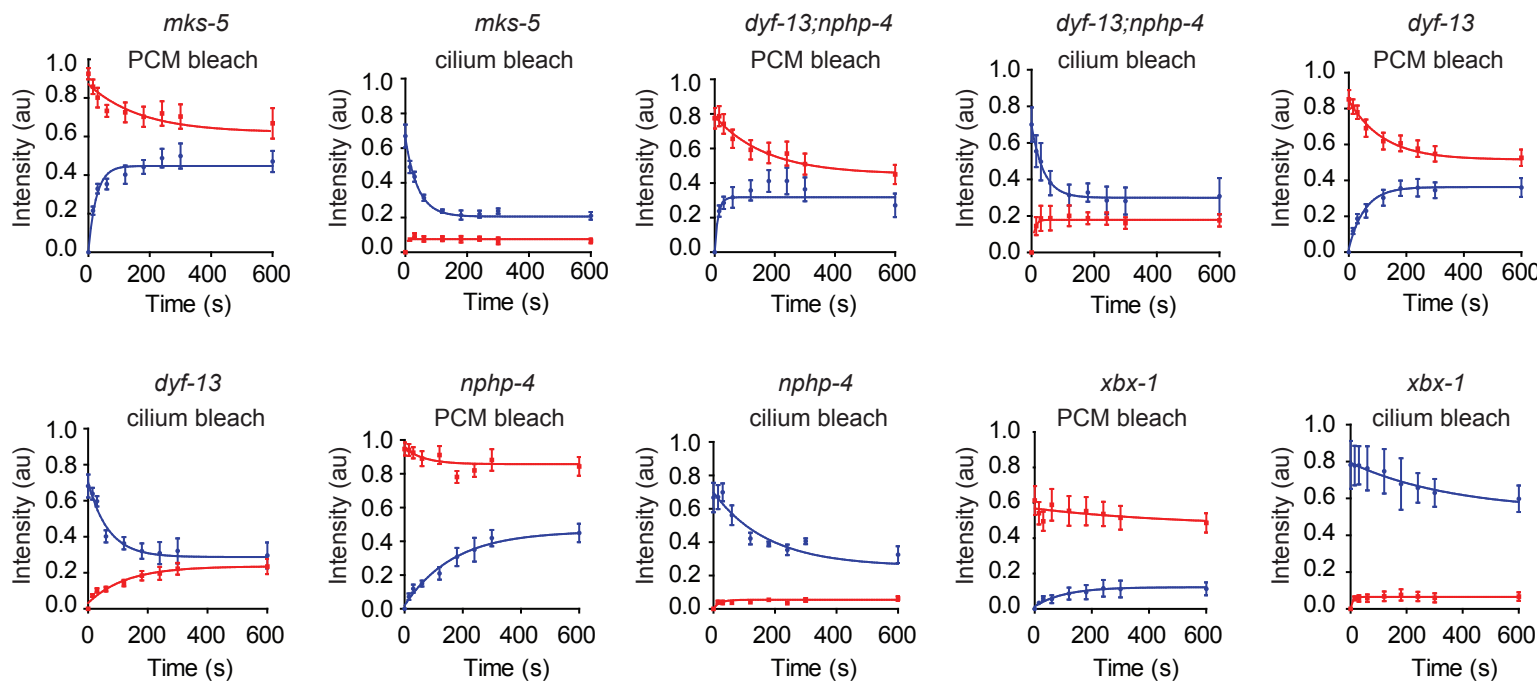**B**

cilium bleach

PCM bleach

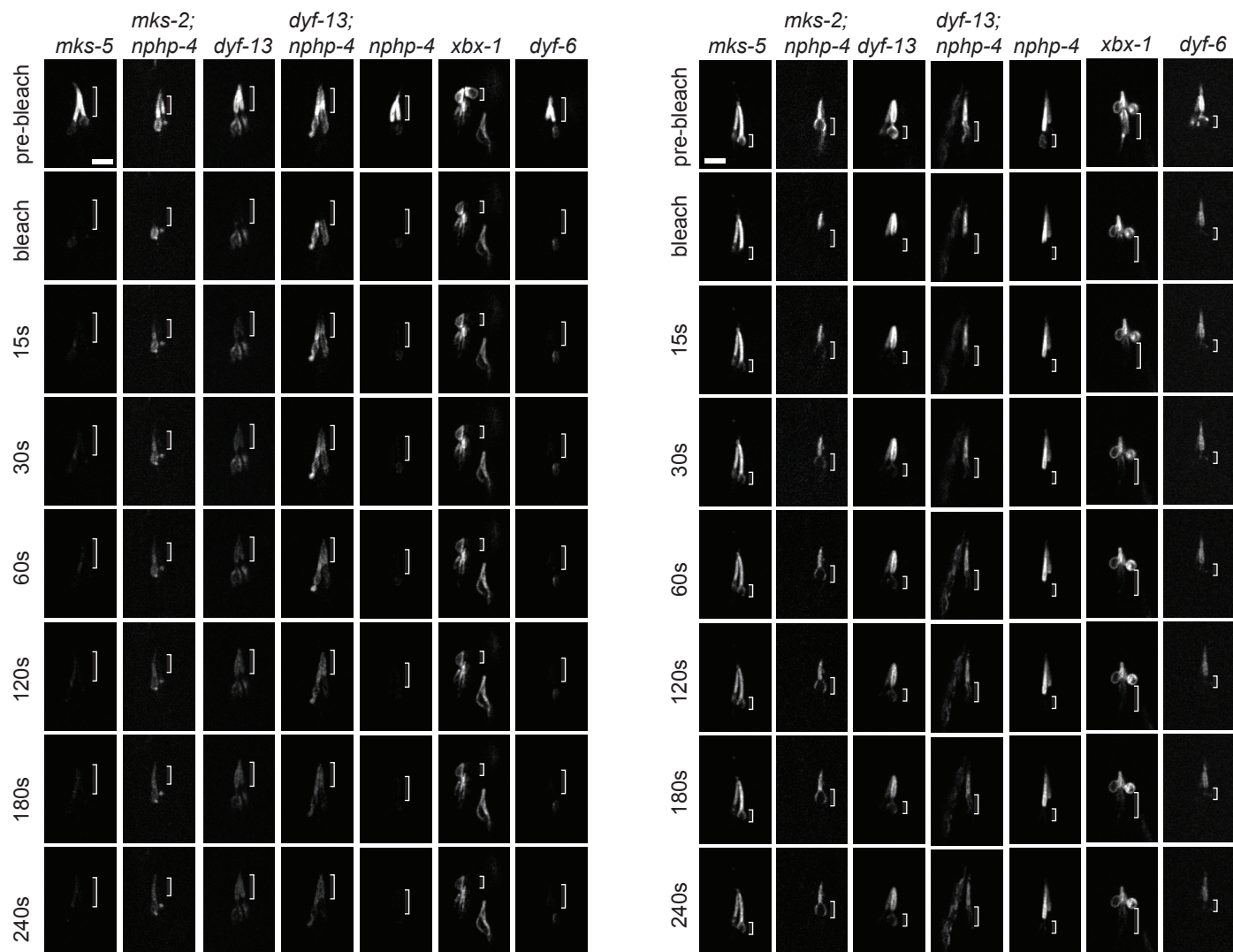

Supplement: Figure S4 — Additional ARL-13 FRAP curves (bleaches of PCM or cilium) and images, linked to Figure 4. (A) Each graph shows the PCM and ciliary FRAP curves. All curves are subtracted for background photobleaching and any very low level recovery from PCM+ ciliary bleaches (shown in Figure 4). Data shows that bleaching of one pool (PCM or cilium) correlates with a signal reduction in the other pool; thus, recoveries come from the non-bleached pool (PCM or cilium). (B) Representative images from phasmid cilia photobleaching experiments. Brackets denote bleached regions. All images identically imaged and scaled. Bars; 2 µm. (PDF) [file pgen.1003977.s004.pdf]

A

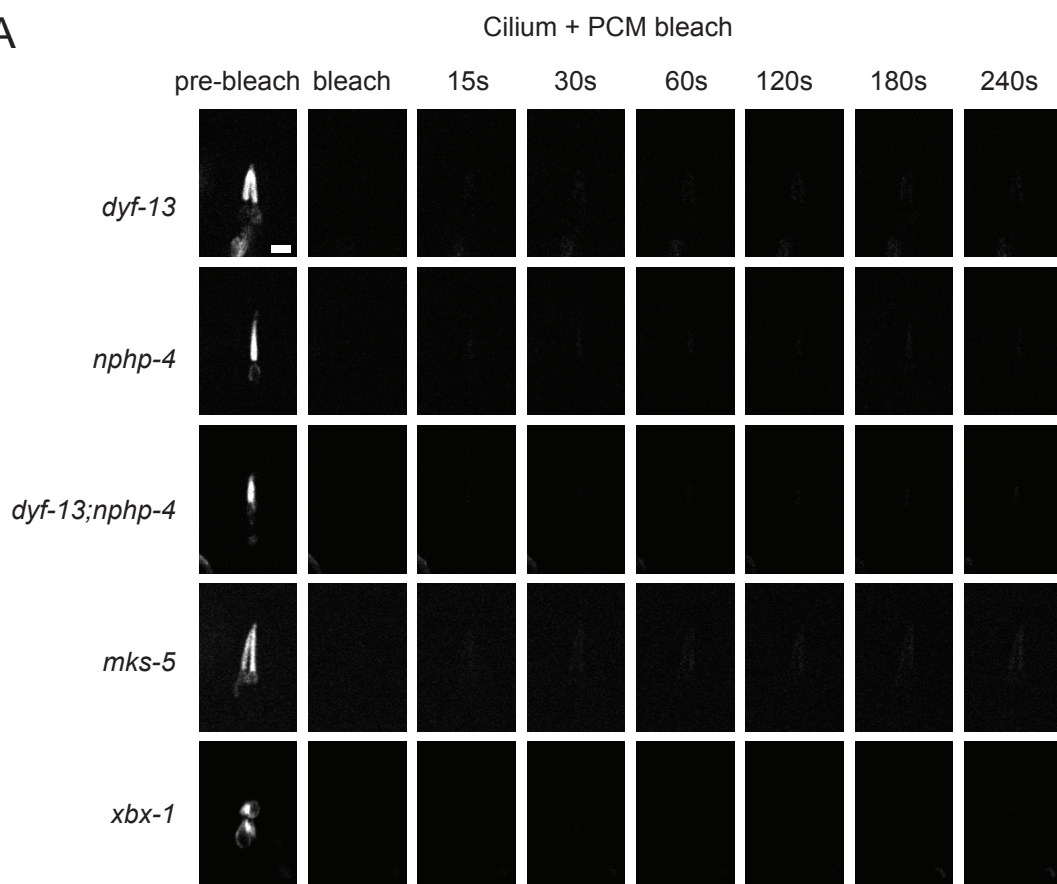

B

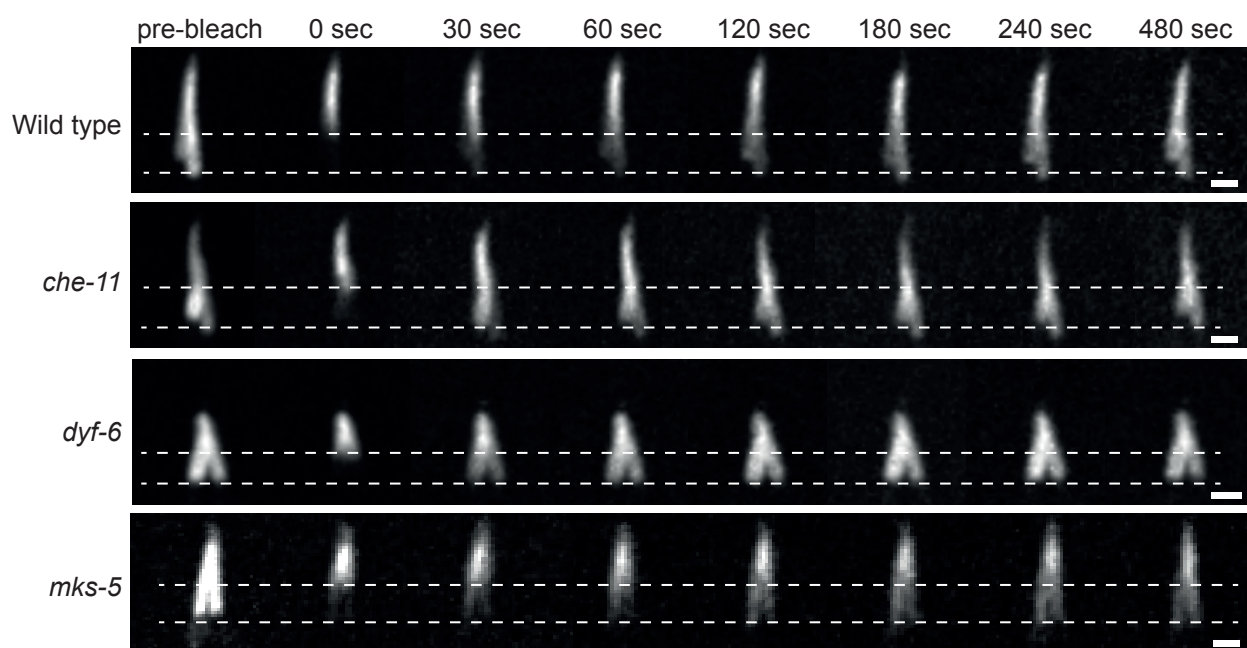

C

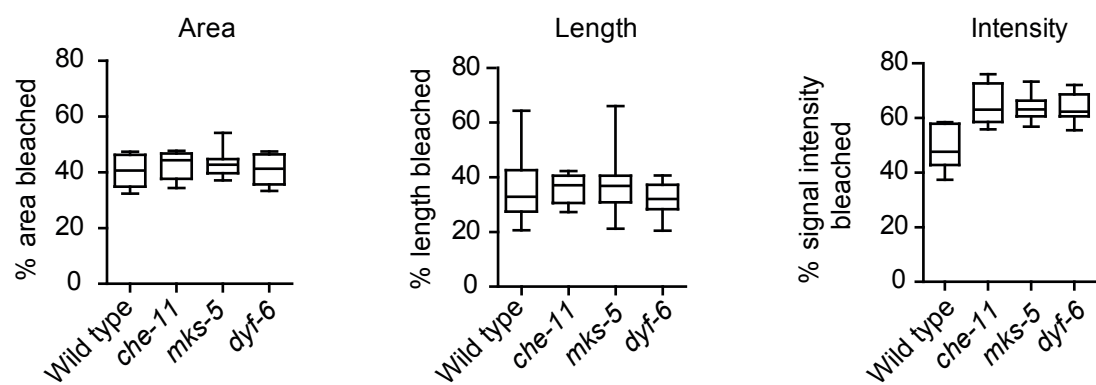

Supplement: Figure S5 — Additional ARL-13 FRAP curves (bleaches of PCM+cilium) and quantitative analysis of bleached region in partial ciliary FRAP experiments, linked to Figure 4. (A) Representative FRAP images after photobleaching entire PCM+ciliary ARL-13 signals in phasmid neurons. Bar; 1 µm. (B) Representative FRAP images after photobleaching ∼40% of proximal-most ARL-13 signals in phasmid cilia. Bars; 1 µm. (C) Box and whisker (min to max) distribution plots showing the % area, length and intensity of photobleached ARL-13::GFP in the partial ciliary FRAP experiments shown in Figure 4C. (PDF) [file pgen.1003977.s005.pdf]

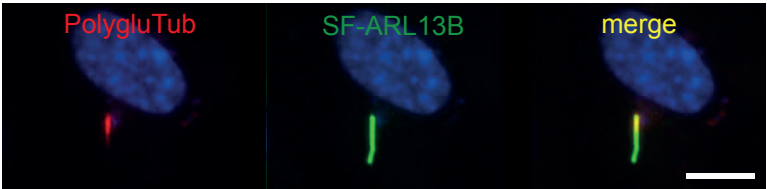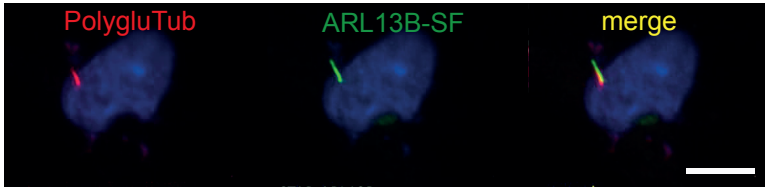

Supplement: Figure S6 — Strep/Flag (SF)-tagged ARL13B localises to hTERT-RPE1 primary cilia, linked to Figure 5. hTERT-RPE1 cells transiently transfected with N-terminally SF (Strep-Flag)-tagged ARL13B or C-terminally SF-tagged ARL13B show specific ciliary localisation of human ARL13B. Green; anti-Flag antibody staining. Red; anti-polyglutamylated tubulin antibody staining. Bars; 10 µm. (PDF) [file pgen.1003977.s006.pdf]

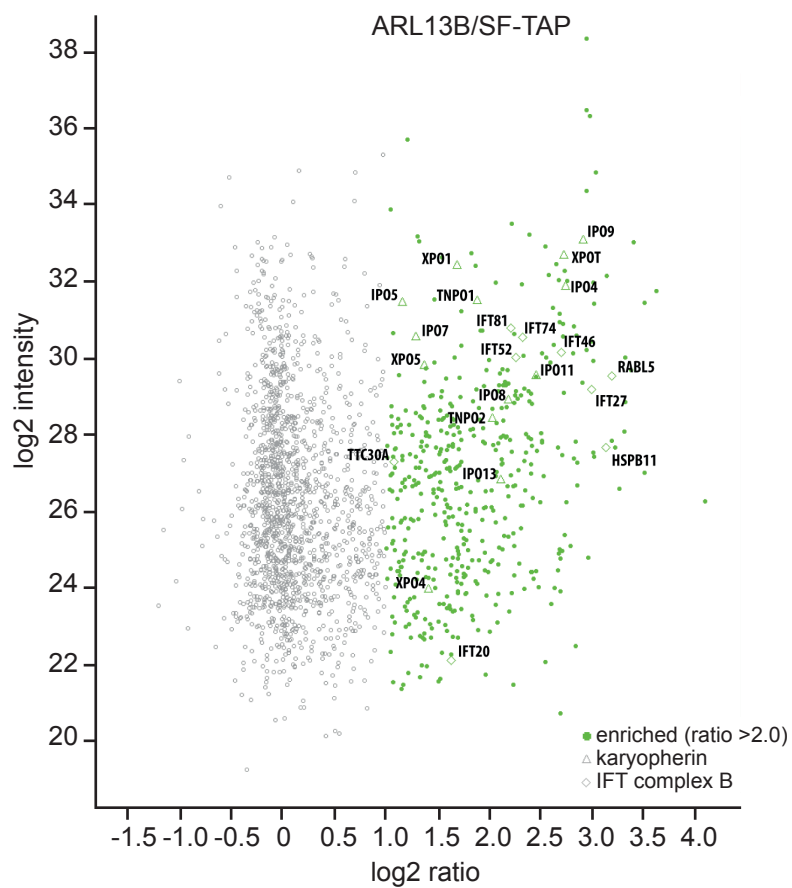

Supplement: Figure S7 — Investigation of ARL13B complexes using SILAC-based quantitative affinity proteomics, linked to Figure 5. Detection of proteins associated with wild type (WT) ARL13B protein complexes in HEK293 cells. Plotted are log2 ratios of proteins enriched in SF-ARL13B(WT) versus SF-control purifications (x-axis) and log2 intensities (y-axis) for each protein identified and quantified in at least two of four biological replicates. Enriched proteins (ratio>2) are plotted in green. (PDF) [file pgen.1003977.s007.pdf]
